# A Fully Biomimetic Flexible Sensor Inspired by the Natural Layered Structure of Eggshells for Multimodal Human–Computer Interaction

**DOI:** 10.1007/s40820-026-02101-2

**Published:** 2026-02-09

**Authors:** Weiwei He, Yanzhen Zhang, Puye Zhang, Yunlong Liu, Guanyang Wu, Boce Xue, Guoqing Hu, Runsheng Li, Chao Zheng, Dongzhi Zhang

**Affiliations:** 1 Shandong Key Laboratory of Design and Manufacturing for High-End Offshore Oil and Gas Equipment, College of Mechanical and Electronic Engineering, China University of Petroleum (East China), Qingdao, 266580 People’s Republic of China; 2 National Engineering Research Center of Marine Geophysical Prospecting and Exploration and Development Equipment, China University of Petroleum (East China), Qingdao, 266580 People’s Republic of China; 3 Aviation Services Research Centre, The Hong Kong Polytechnic University, Hong Kong, 999077 People’s Republic of China; 4 State Key Laboratory of Chemical Safety, College of Control Science and Engineering, China University of Petroleum (East China), Qingdao, 266580 People’s Republic of China

**Keywords:** Eggshell-inspired design, Layered structure, Fully biomimetic sensor, Multimodal sensing, Human–computer interaction

## Abstract

The rapid advancement of naturally microstructure-bioinspired flexible sensors has sparked interest in creating multifunctional systems for human–computer interaction (HCI). However, most existing biomimetic sensors struggle to integrate multiple sensing modes, limiting their practical applications. Herein, this study proposes a design concept for a fully biomimetic sensor. By employing hybrid manufacturing techniques to achieve layer-by-layer biomimicry of the natural layered structure of eggshells, a flexible sensor with multiple sensing modes is developed. The eggshell-inspired multifunctional hybrid flexible sensor (EMHFS) incorporates four functional layers: a triboelectric layer for noncontact sensing, a piezoresistive layer for pressure sensing, and hydrophilic–hydrophobic layers for directional moisture wicking, breathability, and antibacterial properties. The eggshell-inspired structure enables synergistic functionality, allowing seamless switching between contact and noncontact sensing modes. EMHFS demonstrates exceptional performance in multimodal HCI applications, including gesture-controlled robotic hands, wearable unmanned aerial vehicle control systems, and touchless screen password and gesture unlocking, while also exhibiting remarkable sensitivity to weak physiological signals such as breathing and pulse. This fully biomimetic approach offers a novel solution for advanced, flexible, and multifunctional HCI devices.

**Supplementary Information:**

The online version contains supplementary material available at 10.1007/s40820-026-02101-2.

## Highlights

- Drawing inspiration from natural layered structure of eggshells, a fully biomimetic flexible sensor achieves seamless contact–noncontact sensing switching.
- Synergistic integration of triboelectric, piezoresistive, and hydrophilic–hydrophobic layers endows the sensor with exceptional sensitivity, long-term durability, antibacterial activity, and directional moisture management.
- Pioneering multimodal human–computer interaction applications span gesture-controlled robotic hand, wearable unmanned aerial vehicle manipulation, touchless gesture/password unlocking, and high-fidelity monitoring of weak physiological signals.

**Supplementary Information:**

The online version contains supplementary material available at 10.1007/s40820-026-02101-2.

## Graphical abstract

**Figure:**
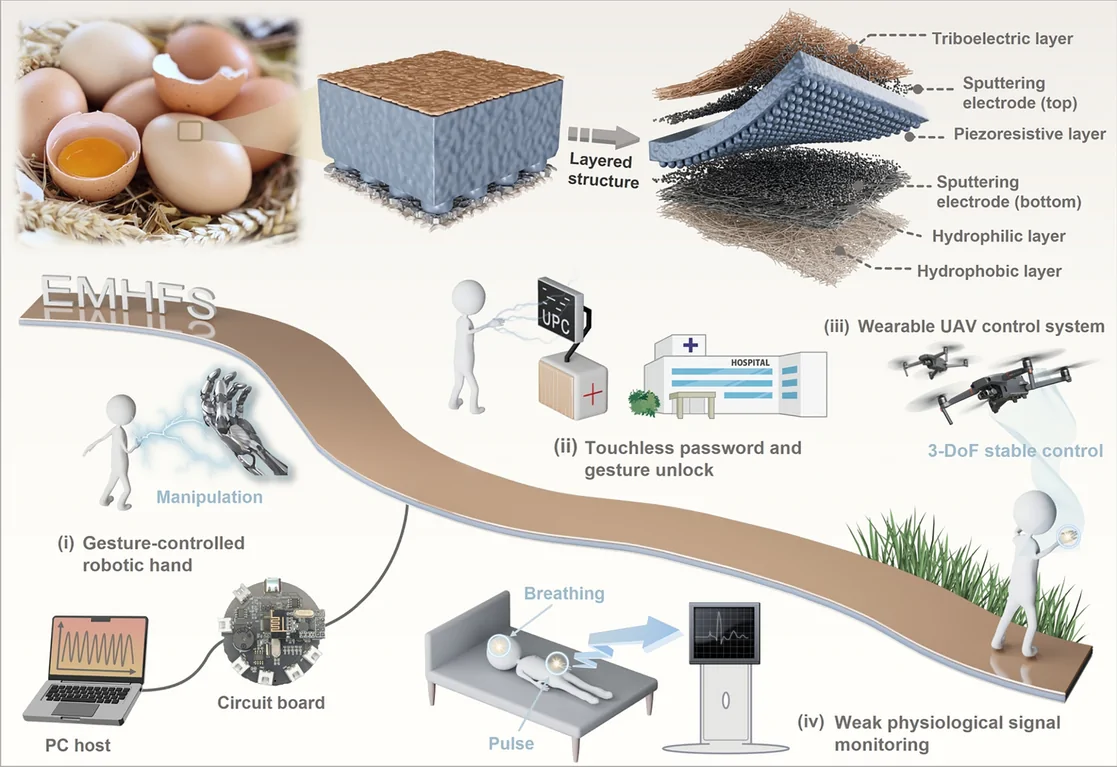

## Introduction

Over millions of years of natural selection, living organisms have evolved intricate structures with unique functions, providing abundant inspiration for designing advanced functional materials and devices. By replicating the structural features and functional mechanisms of organisms, biomimetic engineering has emerged as a powerful strategy to address key challenges in flexible electronics [1–3], especially in the development of high-performance sensors for human–computer interaction (HCI) [4–7]. Flexible sensors based on biomimetic designs have attracted significant attention for their ability to enhance sensor performance by mimicking the mechanical flexibility and structures of biological organisms, showing great potential for applications in wearable health monitoring [8–10], smart robotics [11, 12], and smart interactive devices [13, 14].

However, despite significant research progress, current biomimetic flexible sensors still face notable limitations that hinder their practical application in complex multimodal HCI scenarios. One major bottleneck is the incompleteness of existing biomimetic strategies. Most sensors mimic only a single structural feature of the target organism, resulting in low functional integration and limited sensing modalities. For example, Chen et al. proposed a biomimetic flexible sensor with a multi-level dome structure inspired by octopus tentacles, achieving high sensitivity (0.15 kPa^−1^), high cyclic stability (22,000 cycles at 15 kPa), and a wide pressure sensing range (0–40 kPa) [15]. Inspired by cat whiskers, Xie et al. prepared a biomass hemp fiber/sodium alginate aerogel (BFA) pressure sensor with high sensitivity (6.01 kPa^−1^), fast response time (255 ms), and outstanding durability [16]. Yan et al., inspired by spider webs, developed a flexible pressure sensor featuring high sensitivity, adjustable sensing range (0.5–40 N), and outstanding cyclic stability (5000 cycles) [17]. Other designs adopt multiple biomimetic approaches, integrating structural elements from different biological systems, but typically focus solely on optimizing microstructural combinations, failing to leverage the synergistic effects between multiple natural structures. For instance, Ouyang et al. developed a multifunctional electronic skin for pressure, humidity, and temperature detection by mimicking spider web structures, internal bead chains, and ant antennae [18]. The electronic skin exhibited high pressure sensitivity (0.48 V kPa^−1^), a wide detection range (0–135 kPa), a broad humidity range (25–85% RH), fast response/recovery time (16/25 ms), excellent temperature coefficient of resistance (TCR) (0.0075 °C^−1^), and temperature sensitivity (27–55 °C). Wang et al. proposed a pressure sensor combining sea urchin-like microstructures with a porous honeycomb configuration, achieving high sensitivity (680 kPa^−1^), fast response (10 ms), a wide pressure range (0–150 kPa), and good repeatability (over 3500 cycles at 110 kPa) [19]. He et al., inspired by micro shark spine scales and multi-layer dome structures from crocodile skin, developed a shark–shark (S–S), crocodile–crocodile (C–C), and crocodile–shark (C-S) biomimetic sensors through a proposed cross-scale complementary composite flexible pressure sensor integration strategy [20]. These sensors achieved excellent sensitivity in the low-pressure range (S–S, 32 kPa^−1^), stable deformation at high pressures (C–C, ≈100 kPa), and a wide pressure detection range (C-S, 1 Pa-80 kPa), with fast response/recovery time (21/28 ms), and excellent durability (20,000 cycles). While the multi-biomimetic strategy enables cooperative enhancement of sensor performance, it is still limited to optimizing performance in a single-contact sensing mode and lacks environmental adaptability, making it difficult to meet the diverse demands of smart interactive scenarios. These challenges highlight the urgent need for a fully integrated biomimetic design capable of replicating the multi-level structures with functional synergy in individual natural organisms, achieving multi-mode sensing and environmental adaptability.

As a classic example of natural biomineralization, eggshells feature a well-ordered layered structure with distinct, complementary functions across layers. The outermost cuticle layer acts as a protective barrier, the intermediate spongy and papillary layers collaborate to provide impact cushioning and compressive strength, and the inner and outer shell membranes allow for gas exchange while blocking microbial intrusion [21–23]. This orderly, non-interfering layered structure provides an ideal template for flexible sensor development. Unlike fragmented or multi-combination biomimetic designs, replicating the entire layered structure of an eggshell from top to bottom enables intrinsic functional integration, as the natural synergy between the layers ensures structural compatibility and performance complementarity.

Inspired by this, this study innovatively proposes a fully biomimetic design concept. By replicating the complete layered structure of an eggshell and leveraging hybrid manufacturing techniques, a top-down, fully biomimetic multifunctional flexible sensor is developed. This eggshell-inspired multifunctional hybrid flexible sensor (EMHFS) comprises four functional layers, each corresponding to a key component of the eggshell: (1) The triboelectric layer comprises polyvinylidene fluoride (PVDF)/MXene/BaTiO_3_ nanofibers obtained via electrospinning. This layer mimics the eggshell's cuticle layer, enabling noncontact sensing based on triboelectric effects and electrostatic induction principles. (2) Carbon nanotubes (CNTs) with high aspect ratio and superior electromechanical properties are incorporated as functional fillers into polydimethylsiloxane (PDMS). The PDMS/CNTs piezoresistive layer featuring papillary microstructures is then fabricated via a templating method. This layer mimics the eggshell's spongy and papillary layer, enabling gradient pressure sensing. 3) The tightly bonded hydrophilic–hydrophobic bilayer membrane is formed by combining hydrophilic polyacrylonitrile (PAN) and hydrophobic thermoplastic polyurethane (TPU) nanofibers obtained via continuous electrospinning. This layer, analogous to the inner and outer shell membranes of the eggshell, provides environmental interference resistance and ensures the EMHFS's performance in variable conditions. This fully biomimetic approach replicates the entire hierarchical structure of a single natural organism rather than partial or cross-organism features, enabling seamless integration of contact and noncontact sensing modes. EMHFS can flexibly switch between sensing modes. In contact sensing mode, it demonstrates excellent pressure sensing performance, including high sensitivity (28.72% kPa^−1^), a wide pressure detection range (0–105 kPa), fast response/recovery times (57/42 ms), and excellent durability (13,000 loading–unloading cycles). When switched to noncontact sensing mode, EMHFS exhibits an ultra-wide detection range up to 2 m and stable electrical output. Furthermore, EMHFS demonstrates enhanced environmental adaptability, including good directional moisture wicking, breathability, and antimicrobial properties (92.9% inhibition rate against *E. coli* and 91.2% against *S. aureus*). These characteristics enable it suitable for monitoring weak physiological signals and diverse multimodal HCI applications, such as gesture-controlled robotic hands, wearable unmanned aerial vehicle (UAV) control systems, and touchless screen interactions. This study confirms the superiority of fully biomimetic design based on natural layered structures, establishing a new paradigm for the development of next-generation multifunctional flexible sensors in advanced HCI systems.

## Results and Discussion

### Inspiration and Functional Demonstration of EMHFS

**Fig. 1:**
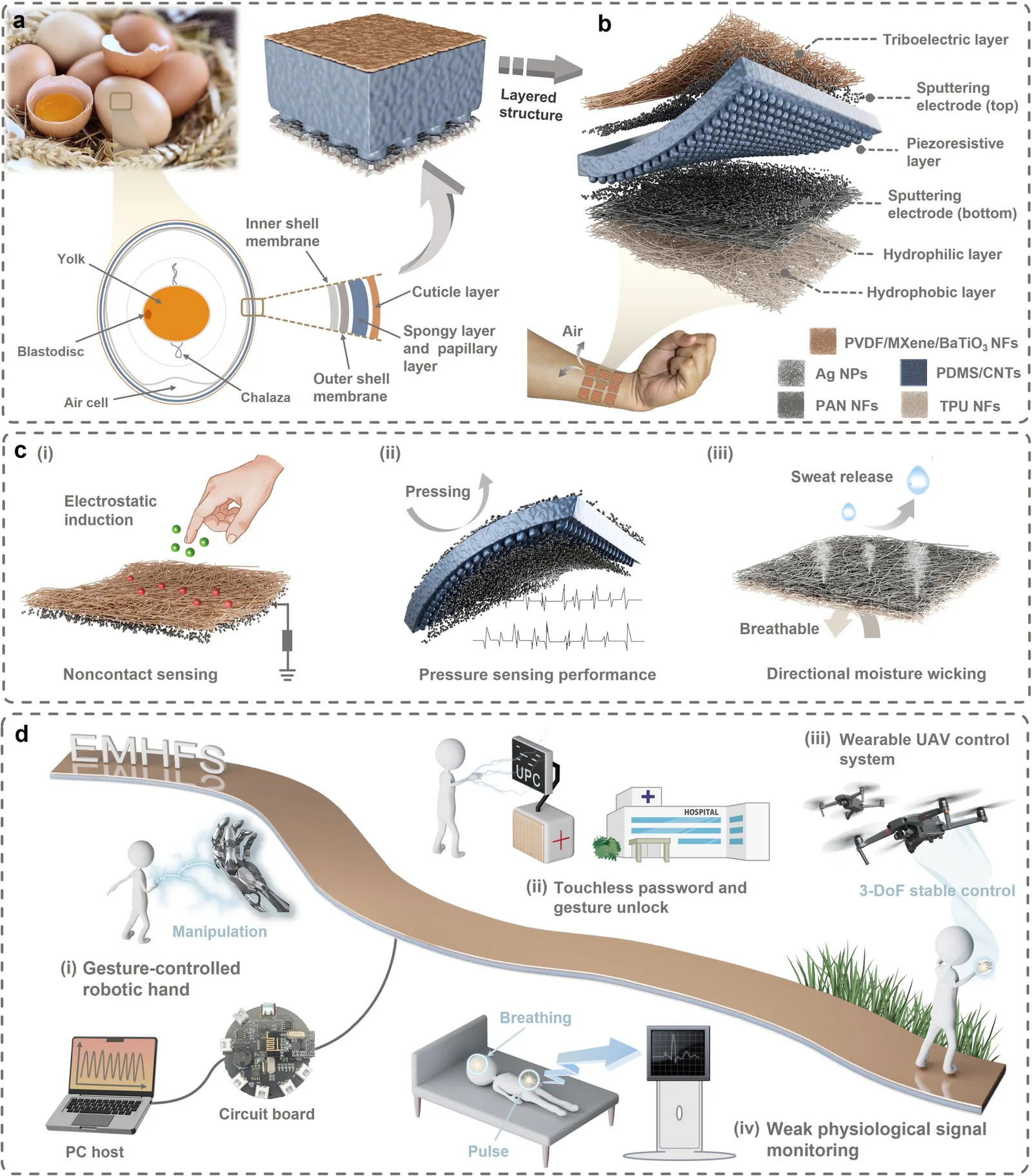
Design inspiration and multifunctional applications of EMHFS. **a** Schematic diagram of egg structure, illustrating the natural layered structure of eggshell. **b** Overall structure of EMHFS. **c** Functional components of EMHFS include (i) noncontact sensing from the triboelectric layer, (ii) pressure sensing in the piezoresistive layer, and (iii) directional moisture wicking of the hydrophilic–hydrophobic layer. **d** Applications of EMHFS across various scenarios include: (i) gesture-controlled robotic hand, (iii) UAV control and (iv) weak physiological signal monitoring in contact mode; as well as (ii) touchless password and gesture unlock in noncontact mode

Eggshells, as typical examples of biomineralization in nature, are not homogeneous structures but instead feature a stratified architecture that serves complementary functions from the exterior to the interior. The distinct layers work synergistically to achieve the core goal of protecting the internal embryo (Fig. 1a). Inspired by the natural layered structure of eggshells, this study developed a top-down fully biomimetic flexible sensor (Fig. 1b). This eggshell-inspired multifunctional hybrid flexible sensor (EMHFS) consists of four parts: a triboelectric layer, a piezoresistive layer, a hydrophilic layer, and a hydrophobic layer. These layers correspond to the cuticle, spongy and papillary layers, outer and inner shell membranes of the eggshell, respectively, each possessing unique functional characteristics without interfering with each other (Table S1).

The triboelectric layer on top of EMHFS is composed of PVDF/MXene/BaTiO_3_ nanofibers obtained via electrospinning. Based on the coupling of triboelectric effect and electrostatic induction, EMHFS achieves noncontact sensing functionality (Fig. 1c(i)). The role of the papillary layer in the eggshell is to buffer external impacts, while the spongy layer provides mechanical strength and compressive resistance. This functional synergy of pressure gradient inspired the design of the piezoresistive layer. The PDMS/CNTs piezoresistive layer with papillary microstructures was fabricated via a mold method. The papillary microstructure sensitively detects minor variations in external pressure, while the spongy-like portion responds to larger applied forces. This design approach endows EMHFS with excellent pressure sensing performance (Fig. 1c(ii)). The outer and inner shell membranes of eggshells consist of densely arranged intertwined fibrous layers that prevent microbial invasion while allowing gas exchange. Similarly, at the bottom of EMHFS, the hydrophilic layer is first formed using electrospun PAN nanofibers, followed by electrospinning a hydrophobic TPU nanofiber layer on top. This creates a tightly bonded dual-fiber membrane that maintains excellent breathability while enabling directional moisture wicking (Fig. 1c(iii)). Antibacterial properties are introduced by magnetron sputtering silver particles onto the upper surface of the hydrophilic layer, which also serves as the lower electrode for the piezoresistive layer. The silver-coated layer at the bottom of the triboelectric layer functions as the upper electrode for pressure sensing and a single electrode for noncontact sensing.

The eggshell-inspired fully biomimetic strategy grants the EMHFS a multifunctional and synergistic layered structure, demonstrating tremendous potential in multimodal gestural human–computer interaction (HCI). Examples include gesture-controlled robotic hand (Fig. 1d(i)) and a wearable unmanned aerial vehicle (UAV) control system (Fig. 1d(iii)) in contact mode, as well as screen password and gesture unlocking (Fig. 1d(ii)) in noncontact mode. Thanks to its integrated design, the EMHFS allows for flexible switch in either contact or noncontact modes, addressing operational demands across various scenarios. Moreover, for applications involving subtle pressure from the human body, EMHFS achieves precise monitoring of weak physiological signals in diverse environments (Fig. 1d(iv)).

### Working Mechanism and Triboelectric Performance of EMHFS

**Fig. 2:**
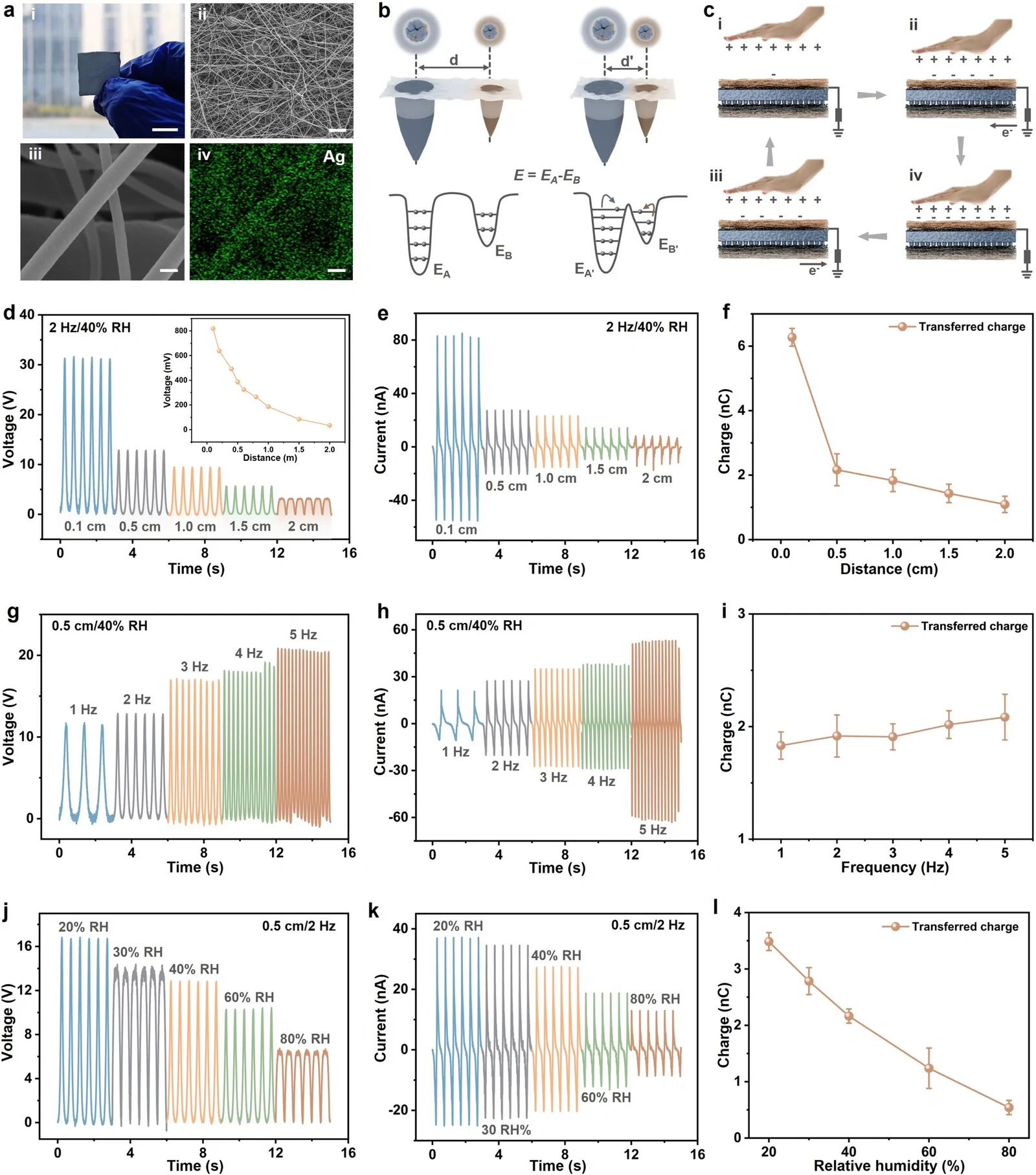
Working mechanism and electrical outputs of EMHFS. **a** (i) Digital image of EMHFS. Scale bars, 1 cm. SEM images of the triboelectric layer: (ii) Top, scale bars, 10 μm. (iii) Bottom, and (iv) the element distribution image of Ag. Scale bars, 200 nm. **b** Electron cloud mode and **c** basic principles for noncontact sensing. **d–f** Influence of distance on the open-circuit voltage, short-circuit current, and transferred charge of EMHFS, respectively, when operated at 2 Hz and relative humidity of 40%. **g–i** Electric outputs of EMHFS under different frequencies from 1 to 5 Hz with 0.5 cm and relative humidity of 40%. **j–l** Effect of relative humidity on the electric outputs of EMHFS at 2 Hz and 0.5 cm

The optical photograph of the EMHFS assembled with adhesive gel in its normal state is shown in Fig. 2a(i). The manufacturing process ensures that the EMHFS exhibits excellent flexibility (Fig. S6). A comparison of the cross-sectional morphology of the eggshell and the EMHFS highlights the structural consistency between the two (Fig. S7). Noncontact sensing in EMHFS is primarily achieved through the friction layer. As the matrix of the triboelectric layer, PVDF possesses excellent piezoelectric and triboelectric properties due to its polar β-phase crystal structure. It serves as the core material for charge generation during noncontact interactions, providing a stable structural framework for the composite nanofibers while facilitating electron transfer at the interface. With high electrical conductivity, large specific surface area, and good compatibility with PVDF, MXene enhances the charge carrier density of the triboelectric layer. It reduces the interface potential barrier between the sensor and approaching objects, accelerates charge separation and transfer, and improves the electrical output intensity and response speed of noncontact sensing. As a high-dielectric ceramic nanofiller, BaTiO_3_ modulates the dielectric properties of the PVDF matrix. It strengthens the local electric field at the interface, further lowering the potential barrier for electron transfer during noncontact interactions. Additionally, BaTiO_3_ improves the mechanical stability of the nanofibers and enhances the triboelectric layer's durability under repeated use. Through systematic optimization experiments, the optimal content of MXene (0.03 g) and BaTiO_3_ (0.1 g) in the friction layer was determined. Specifically, when MXene content is excessively high, agglomeration occurs within the PVDF fiber network, leading to a reduction in pore structure and a weakening of the triboelectric output [24]. When the content is less than 0.03 g, the conductivity of the triboelectric layer is insufficient to support effective charge transfer [25]. For BaTiO_3_, a content of 0.1 g achieves an optimal balance between enhancing the dielectric properties of PVDF and maintaining fiber uniformity. Higher contents lead to fiber breakage during electrospinning, while lower contents fail to significantly improve triboelectric performance [26].

**Fig. 3:**
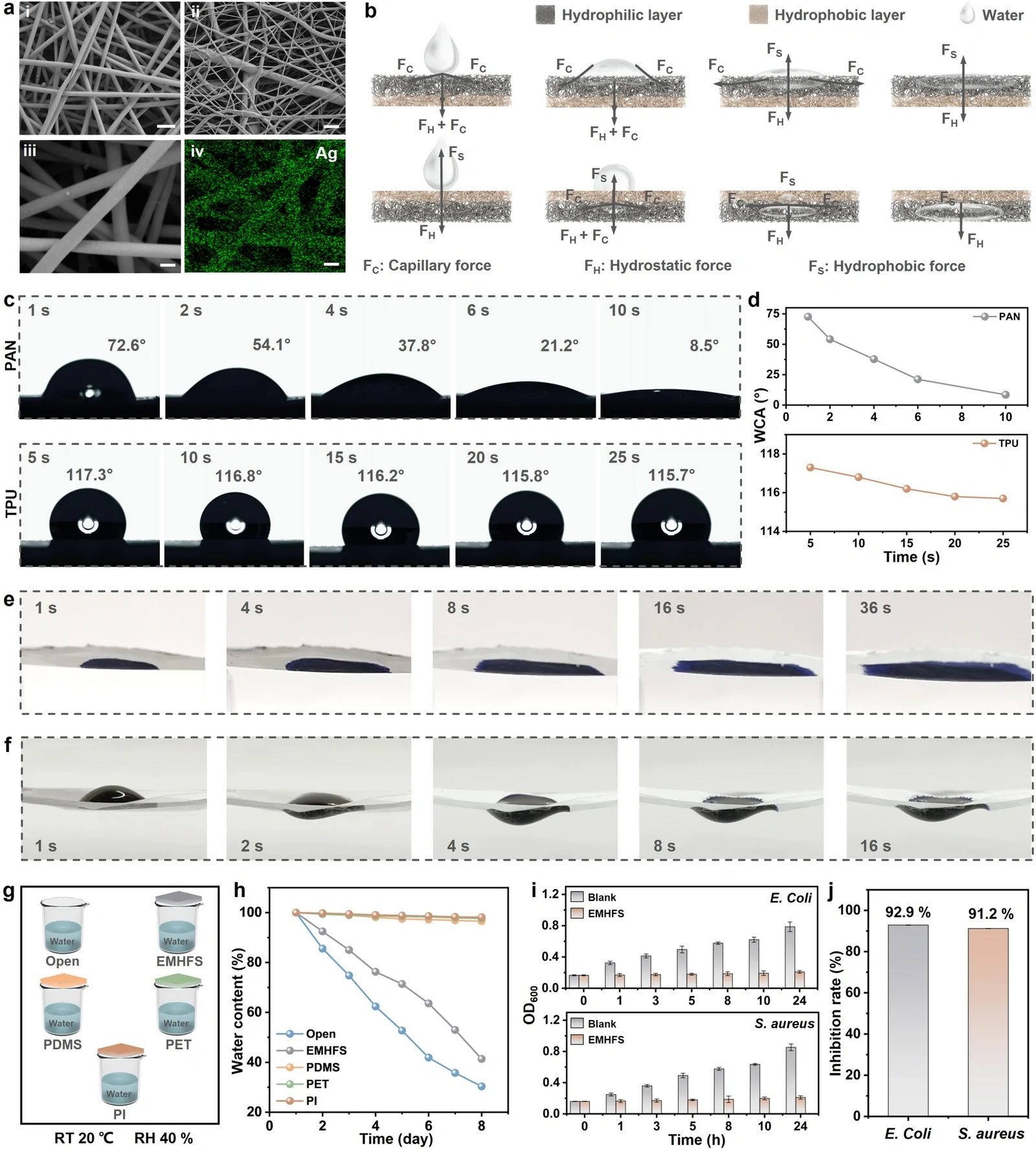
Directional moisture wicking, breathability, and antibacterial properties of EMHFS. **a** SEM images of (i) PAN nanofibers layer. Scale bars, 1 μm. (ii) TPU nanofibers layer. Scale bars, 4 μm. (iii) PAN-Ag electrode, and (iv) the element distribution image. Scale bars, 300 nm. **b** Proposed the directional moisture wicking mechanism of EMHFS form the hydrophobic and hydrophilic layer. **c, d** Time-dependent water contact angle of nanofibers. Macroscopic optical images of the apparent water transport process on **e** hydrophilic PAN nanofibers and** f** hydrophobic TPU nanofibers.** g** Schematic diagram and **h** test curve of breathability experiments for different materials. **i** Optical density versus time curve of *E. coli* and *S. aureus* after co-culturing with blank and EMHFS samples.** j** Antibacterial rates of EMHFS against *E. coli* and *S. aureus* after 24 h of co-cultivation

The morphology of the triboelectric layer composed of PVDF/MXene/BaTiO_3_ nanofibers at the optimal content is shown in Fig. 2a(ii). MXene and BaTiO_3_ are uniformly distributed within the PVDF fiber network, preserving the porous structure of the triboelectric layer, as confirmed by the corresponding element maps (Fig. S8). Due to the excellent hydrophobicity of PVDF fibers, they can suppress signal fluctuations caused by changes in environmental humidity [27]. Simultaneously, the incorporation of MXene mitigates the impact of electromagnetic interference in the environment on the signal, thereby preventing external interference from affecting the noncontact sensing system [28]. Silver nanoparticles were successfully deposited on the bottom of the triboelectric layer using magnetron sputtering, resulting in a compact and uniform silver film (Fig. 2a(iii), (iv)). Identical result was observed on the surface of the hydrophilic PAN layer (Fig. 3a(iii), (iv)). To evaluate electrode reliability during practical use, the surface resistance of both electrodes was measured (Fig. S9). The sheet resistances of the bottom electrode of the triboelectric layer and the surface electrode of the hydrophilic layer were 5.4 and 9.2 Ω sq^−1^, respectively, meeting the demand for efficient conductivity during use. Additionally, the stability of silver layer adhesion and resistance changes after repeated bending and loading was tested (Fig. S10). After 3,000 bending cycles, the surface resistance of the silver layer showed negligible change, indicating that the electrodes maintained sufficient conductivity and integrity after testing.

The working mechanism of EMHFS in noncontact sensing mode is based on the coupling of triboelectric effects and electrostatic induction, enabling the conversion of mechanical motion into electrical signals. The electron cloud potential well model is used to explain the electron transfer phenomenon during the approach process. As shown in Fig. 2b, the distance between two materials with different electronegativities is initially d, where the electron clouds of surface atoms remain independently distributed, with atomic spacing within the range of van der Waals forces, but not entering the repulsion zone. At this point, the potential barrier between the electron clouds is high, hindering electron transfer [29]. When the two material approaches, the distance between surface atoms decreases to d', and the interface potential barrier is lowered to a penetrable range, facilitating electron transfer, thereby generating an electrical output signal [30, 31]. Specifically, the detailed working mechanism of EMHFS is illustrated in Fig. 2c. When the hand approaches the triboelectric layer, electrons within the layer are attracted due to the triboelectric effect [32]. The inherent triboelectric properties of the material enable charge transfer and redistribution. This results in free electrons flowing from the ground to the electrode at the bottom of the triboelectric layer (steps (i-ii)) [33]. As the hand continues to approach the triboelectric layer, interaction between the hand and the layer persists. Based on electrostatic induction, the positive charges of the hand fully shield the negative charges on the surface of the triboelectric layer, reaching electrostatic equilibrium (steps (ii-iii)). Conversely, as the hand moves away, charges dynamically adjust during separation to achieve electrical neutrality, with negative charges flowing back from the triboelectric layer to ground (steps (iii-iv)). When the hand returns to its initial state, the charge distribution in the entire system gradually returns to equilibrium, completing a "contact–induction–separation–recovery" cycle (steps (iv-i)).

The electrical output performance of an EMHFS unit operated by a human finger in noncontact mode was quantitatively analyzed. Figure 2d-f shows the open-circuit voltage, short-circuit current, and transferred charge of the EMHFS at a motion frequency of 2 Hz and 40% relative humidity (RH). All electrical outputs decreased significantly as the distance between the finger and EMHFS increased. With the distance increased from 0.5 to 2 cm, the average peak voltage decreased from 30.25 to 3.14 V, the current decreased from 82.62 to 6.91 nA, and the transferred charge decreased from 6.27 to 1.09 nC. The inset in Fig. 2d shows that the EMHFS can detect a maximum distance of 2 m, with a peak voltage of 34 mV. Figure 2g–i tests the impact of motion frequency on EMHFS's output performance at a distance of 0.5 cm and 40% RH. The results indicated that both voltage and current increase with rising motion frequency. At 5 Hz, the output voltage and current reached 20.37 V and 48.84 nA, respectively. The transferred charge exhibited only a small variation (Δ*C* = 0.25 nC) across different motion frequencies, ensuring the reliability of EMHFS for distance detection [34]. Additionally, the effect of RH on EMHFS's performance was explored at a distance of 0.5 cm and a motion frequency of 2 Hz (Fig. 2j–l). As RH increased from 20% to 80%, the average peak voltage decreased from 16.62 to 6.34 V, the corresponding current dropped from 36.51 to 12.96 nA, and the transferred charge decreased from 3.49 to 0.54 nC. In the daily environments of this study, RH consistently remained around 40% (the human comfort range spans 40–60% RH), indicating that the EMHFS can provide sufficient response in its operational environment.

To further investigate the power output performance of the EMHFS, its voltage, current, and output power density were evaluated under a series of external resistances. As shown in Fig. S11a, the output voltage of the EMHFS initially remained at a minimum value before rising sharply as the resistance increased from 0.1 kΩ to 10 GΩ. In contrast, the output current of the EMHFS gradually decreased with increasing resistance. Furthermore, the instantaneous peak power density of the EMHFS was calculated as $P={U}^{2}/RS$, where *P*, *U*, *R*, and *S* represent the power density, output voltage, external resistance, and active area of the EMHFS, respectively. As shown in Fig. S11b, the instantaneous power density of the EMHFS reached a peak of 5.2 μW cm^−2^ at an external load resistance of 20 MΩ. The stability of the triboelectric output from the EMHFS directly impacts the stability of noncontact sensing. Therefore, the operational endurance of EMHFS was tested to assess its practicality at a frequency of 2 Hz, a noncontact distance of 0.5 cm, and 40% RH. As shown in Fig. S12, after exceeding 12,000 noncontact separation cycles, the EMHFS output voltage remained nearly constant at 12.3 V, demonstrating exceptional reliability. Additionally, comparing the output voltage of the EMHFS in its original state with after compression and torsion reveals that its output voltage remains virtually unchanged, demonstrating exceptional mechanical robustness. The detection range to sensor area (D/S) ratio is calculated as the maximum noncontact detection distance divided by the effective sensing area, which is used to evaluate the noncontact sensing performance per unit area of the sensor. As presented in Table S2, the D/S ratio of EMHFS can reach 88.89, which is the highest among previously reported noncontact sensors, further demonstrating its outstanding noncontact sensing performance.

### Directional Moisture Wicking, Breathability, and Antibacterial Properties of EMHFS

Figure 3a(i, ii) depicts the morphologies of the hydrophilic PAN nanofiber layer and the hydrophobic TPU nanofiber layer, respectively. The nanofibers overall exhibit a randomly distributed nonwoven fabric morphology. In terms of fiber diameter, the PAN layer demonstrates a more uniform fiber size. Furthermore, the pore diameter increases progressively from the PAN layer to the TPU layer, exhibiting a clear pore gradient. To evaluate the directional water transport performance of EMHFS, the wettability of the hydrophilic and hydrophobic nanofiber membranes was first investigated. Figure 3c and d presents the optical images and changes in the water contact angle (WCA) on the nanofiber layer surfaces over time. On the PAN nanofiber layer surface, the WCA decreased dynamically from an initial 72.6° to 8.5° within 10 s, demonstrating the excellent hydrophilicity of the PAN nanofibers. In contrast, the TPU nanofiber layer exhibited stable hydrophobicity, with an initial WCA of 117.3°, showing almost no spreading behavior over 25 s. The water transport capability of the membrane was then monitored through the dynamic transfer process of water droplets across both hydrophilic and hydrophobic sides. As shown in Fig. 3e, when a water droplet contacts the PAN nanofiber layer, it rapidly penetrates and spreads, achieving complete wetting within 36 s. The presence of the hydrophobic layer prevents the droplet from penetrating through the hydrophilic layer and dropping off. When the membrane is inverted and the droplet contacts the TPU nanofiber layer, it penetrates into the hydrophilic layer within 16 s, eventually achieving fully wetted or even drops off (Fig. 3f). These results demonstrate that tightly bonded hydrophilic and hydrophobic layers obtained through layer-by-layer spinning can unidirectionally transport water droplets from one side of the membrane to the other.

In fact, the directional moisture-wicking capability of EMHFS is achieved through the push–pull effect induced by the fiber pore gradient and hydrophilic–hydrophobic differences. In this study, we comprehensively analyzed the working principle of directional moisture wicking and the key parameters influencing its functionality, from macroscopic forces to microscopic pressure differences. Specifically, as shown in Fig. 3b, when a water droplet falls on the upward-facing hydrophilic layer surface, the strong hydrophilicity of the layer generates capillary forces (*F*_C_) acting in all directions except upward. Simultaneously, due to the droplet's own gravitational force, it experiences a downward hydrostatic pressure (*F*_H_). The combined effect of *F*_C_ and *F*_H_ drives water penetration into the hydrophilic layer. When the droplet spreads to the hydrophilic–hydrophobic interface, the hydrophobic force (*F*_S_) generated by the hydrophobic layer creates an upward repulsive force. This repulsive force from the hydrophobic layer limits the penetration of the droplet into the hydrophobic layer, forcing it to stay directed within the hydrophilic layer. Conversely, when the hydrophobic layer faces upward and the droplet contacts the surface, it experiences an upward force (*F*_S_) and a downward force (*F*_H_). As droplet numbers increase, the combined force of *F*_S_ and *F*_H_ drives droplets downward toward the hydrophilic layer. When droplets breach the hydrophobic layer's pores and contact the hydrophilic layer, the direction of *F*_S_ reverses, and together with *F*_C_ from the hydrophilic layer pull droplets inward until fully absorbed or even drops off. The synergistic interaction of *F*_S_ and F_C_ generates a push–pull effect, constituting the macroscopic driving force for the directional motion of the droplet. The magnitude of *F*_C_ can be derived using the Young–Laplace equation, expressed as [35]:

1 $${F}_{\mathrm{C}}=2\pi r\gamma \mathrm{cos}\theta$$

where *r*, *γ*, and *θ* represent the radius of the nanofiber pore, the surface tension of the water droplet (approximately 72 mN m^−1^ at room temperature), and the contact angle of the droplet on the fiber surface, respectively. The hydrophilic layer exhibits a contact angle *θ* < 90°, causing the *F*_C_ to attract water droplets. Conversely, the hydrophobic layer presents a contact angle *θ* > 90°, resulting in a repulsive force against water droplets. This difference in push–pull forces due to hydrophilic–hydrophobic contrast ultimately leads to the directional moisture wicking and unidirectional transport of water droplets. Additionally, *r* is another key parameter that determines the capillary force. The hydrophilic layer features denser nanofiber pores (smaller *r*), resulting in higher capillary force density per unit area and enhanced hydrophilic properties. Conversely, the hydrophobic layer's coarser pores (larger *r*) increase capillary forces (repulsion) acting on water droplets, yielding enhanced hydrophobic properties. Thus, the design of the pore gradient further promotes directional moisture wicking of water droplets. The distribution density of *F*_C_ per unit contact area can be quantified by capillary pressure (*P*_C_). For a single nanofiber pore, the corresponding pore contact area (*A* = *πr*^*2*^) allows derivation of the *P*_C_ calculation equation [36]:

2 $${P}_{\rm C}=\frac{{F}_{\rm C}}{A}=\frac{2\gamma \mathrm{cos}\theta }{r}$$

When the water droplet contacts the fiber surface, a liquid–solid–gas three-phase contact line (TCL) forms at the intersection of the droplet edge, air, and the fiber surface. The capillary pressure causes the TCL to shift between hydrophobic nanofibers, forming a convex curved surface. The shape of the TCL directly reflects the wettability of the fiber surface. The additional capillary pressure exerted on the droplet at this curved surface is quantified by calculating the pressure difference at the TCL. The equation for the capillary pressure difference (∆*P*) is as follows [37]:

3 $$\Delta P=\frac{\gamma }{{R}^{\prime}}=\frac{2\gamma {\sin}\left[\theta \left(\Psi \right)-\Psi \right]}{r+2{R}_{0}\left(1-{\sin}\Psi \right)}$$

where *R'*, *R*_0_, *Ψ*, and *θ(Ψ)* represent the curvature radius of the surface, the nanofiber radius, the local geometric angle, and the contact angle of the water droplet on the fiber surface (which varies with the geometric angle *Ψ*), respectively. At the microscopic scale, we comprehensively analyzed the key parameters influencing directional moisture wicking by constructing a hydrophilic–hydrophobic nanofiber model (Fig. S14). When the hydrophilic layer is on top, ∆*P* manifests as an upward force due to the attraction of the hydrophilic layer. This force balances the downward hydrostatic pressure *F*_H_, keeping the droplet stably within the hydrophilic layer and preventing it from permeating into the hydrophobic layer. When the hydrophobic layer is on top, ∆*P* exhibits an upward repulsive force due to the repulsive effect of the hydrophobic layer. This force synergizes with the downward hydrostatic pressure *F*_H_, propelling the water droplet through the pores of the hydrophobic layer toward the underlying hydrophilic layer. It is important to note that water droplets only begin to contact and diffuse into the hydrophilic layer when the height of the curved protrusion (*h*) equals the distance between hydrophilic and hydrophobic nanofibers (*H*). Therefore, the hydrophilic nanofibers should have a larger thickness (reducing *H*) to facilitate directional water transport while storing more moisture.

Similar to the outer and inner eggshell membranes allowing for gas exchange, the EMHFS with multilayered stacked nanofiber membranes, which also possess a porous structure, exhibits excellent breathability and moisture permeability. As shown in Fig. S15, the EMHFS is fixed in a clamp, and when air is pumped into the water, the gas passes through the sensor and forms numerous bubbles. Subsequently, the moisture permeability of the EMHFS was investigated. The EMHFS, polydimethylsiloxane (PDMS) film, polyethylene terephthalate (PET) film, and polyimide (PI) film were each placed over beakers filled with water, and the changes in water content over a week were monitored at room temperature and 40% RH (Fig. 3g). An uncovered beaker served as a control. As shown in Fig. 3h, both the control and EMHFS exhibited continuous water loss over time, while the water content in the beakers covered with PDMS, PET, and PI films remained nearly constant. These results demonstrate the excellent moisture permeability of EMHFS. Furthermore, the silver layer on the hydrophilic PAN nanofibers not only serves as an electrode but also confers significant antibacterial properties to the EMHFS. To evaluate its antimicrobial activity, Gram-negative Escherichia coli (*E. coli*) and Gram-positive Staphylococcus aureus (*S. aureus*) were used as pathogenic bacteria and tested via the plate colony count method. As shown in Fig. S16, EMHFS exhibited significant inhibitory effects against both bacteria compared to the blank control group. The changes in viable bacterial concentration were monitored via optical density (OD) values for both strains (Fig. 3i). Over time, the bacterial concentrations in the blank control group increased continuously, whereas OD values of EMHFS remained nearly constant. After co-culturing for 24 h, EMHFS achieved antibacterial rate of 92.9% against *E. coli* and 91.2% against *S. aureus* (Fig. 3j). The significant antibacterial activity of EMHFS can be attributed to silver nanoparticles in the silver layer, which penetrate bacterial cells and bind with thiol (–SH) groups in enzymes, disrupting bacterial structures and ultimately causing bacterial death [38]. The antibacterial rate is calculated using equation [39]:

4 $$\text{Antibacterial rate}=\left(1-\frac{\mathrm{OD}_{\rm e1}-\mathrm{OD}_{\rm e0}}{\mathrm{OD}_{\rm b1}-\mathrm{OD}_{\rm b0}}\right)\times 100\%$$

where OD_e0_ and OD_b0_ represent the initial bacterial concentrations in the experimental group and blank control group, respectively. OD_e1_ and OD_b1_ represent the bacterial concentrations in the experimental group and blank control group after co-culture, respectively.

### Pressure Sensing Mechanism and Performance of EMHFS

**Fig. 4:**
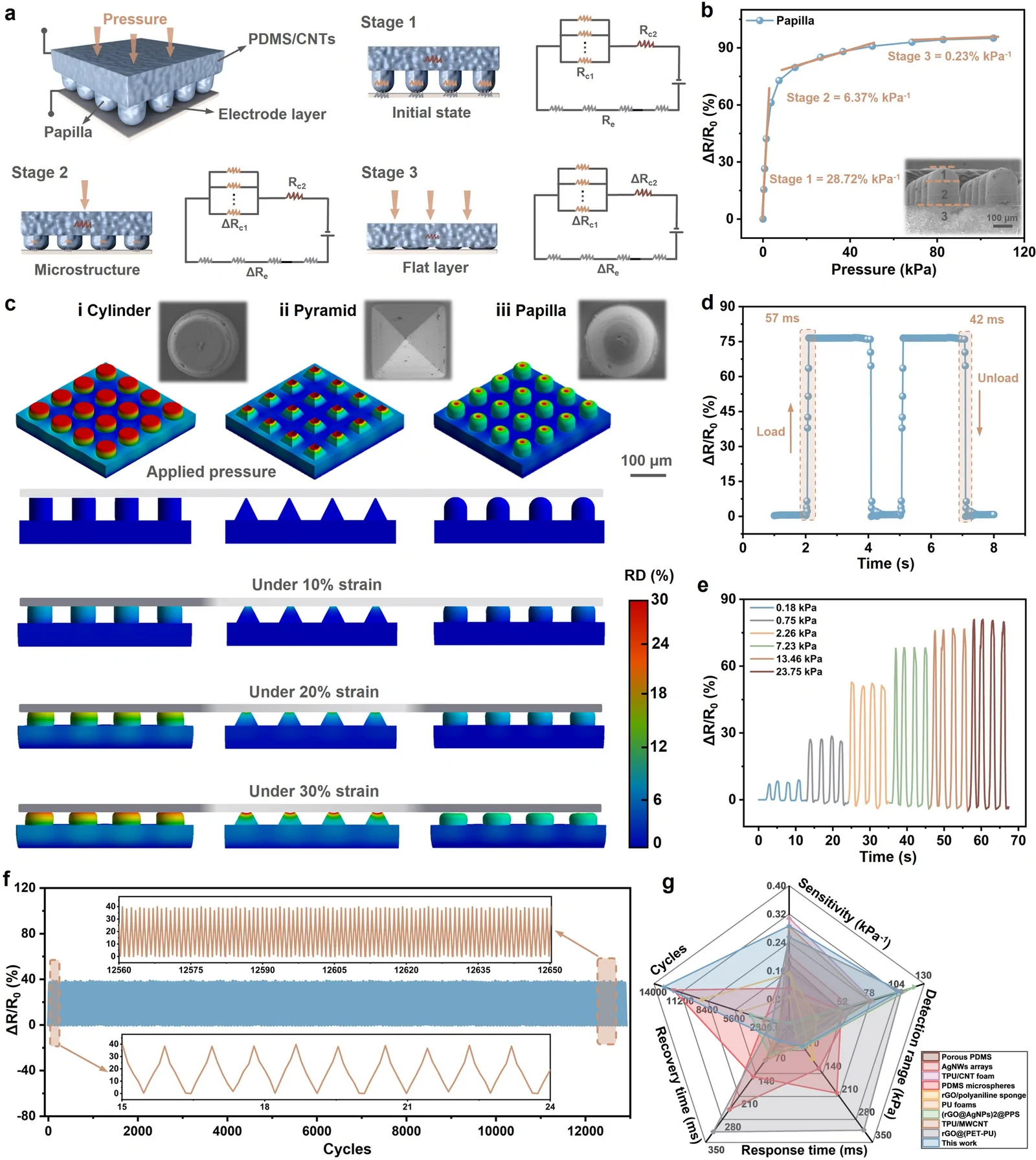
Pressure sensing mechanism and performance of EMHFS.** a** Sensing mechanism of the papillary microstructure on the piezoresistive layer of EMHFS and its corresponding equivalent circuit diagram. **b** Sensitivity curve of EMHFS. **c** FEA simulates the compressive deformation behavior of (i) cylindrical, (ii) pyramidal, and (iii) papillary microstructures under loading conditions and their corresponding models. **d** Response and recovery time of EMHFS under 10 kPa pressure. **e** Response curve of EMHFS under different pressures. **f** Cyclic stability test of EMHFS for 13,000 repeat cycles under 1.5 kPa. **g** Comparison of EMHFS performance with previous research

In contact mode, the pressure sensing performance of EMHFS depends on the design of the piezoresistive layer. The design concept draws inspiration from the spongy and papillary structures of eggshells. Using a templating method, papillary microstructures were fabricated on the surface of the flat layer, expanding the pressure detection range while achieving high sensitivity. The papillary microstructure provides a compressible morphology when subjected to minute pressures, serving as the key factor for achieving high sensitivity. The flat layer, analogous to the spongy layer in eggshells, can withstand higher pressures and respond accordingly, determining the upper limit of pressure detection for the EMHFS. Figure 4a illustrates the pressure sensing principle of EMHFS and establishes an equivalent circuit to describe the resistance contributions. The total resistance can be calculated as follows:

5 $$R={\sum }_{i=1}^{m}\frac{1}{{R}_{\rm c1i}}+{R}_{\rm c2}+{\sum }_{j=1}^{n}{R}_{\rm ej}$$

where *m*, *n*, *R*_c1i_, *R*_c2_, and *R*_ej_ represent the number of papillary microstructures, the number of surface contacts between fibers, the contact resistance between single papillary structure and the electrode, the internal resistance of the flat layer, and the surface contact resistance between silver-plated nanofibers, respectively. To simplify analysis, the total contact resistance of the parallel-connected papillary structures is denoted as* R*_c1_, while the conductive pathways between fibers are connected in series as a total resistance *R*_e_. The simplified equation becomes:

6 $$R={R}_{\rm c1}+{R}_{\rm c2}+{R}_{\rm e}$$

Based on the magnitude of external pressure applied to the EMHFS, the response process can be specifically divided into three stages. In the initial state, the papillary microstructures are in point contact with the conductive nanofibers, resulting in a limited contact area and high resistance values for *R*_c1_ and *R*_c2_ under no-load conditions. The fiber electrodes maintain their inherent resistance *R*_e_, resulting in a relatively high total resistance. When a small external force is applied, the papillary microstructure undergoes compression deformation. This increases the contact area between the papillary tips and the fiber electrodes, and the rise in parallel resistance causes the contact resistance *R*_c1_ to decrease. Conversely, the internal resistance *R*_c2_ remains largely unchanged, as minor pressure is insufficient to deform the internal conductive network of the flat layer. In fact, the applied pressure reduces the surface contact resistance *R*_e_ between fiber electrodes, which is related to the electrode fabrication process. Specifically, the electrodes in this study were formed by magnetron sputtering silver nanoparticles onto PAN nanofibers. On a microscopic scale, the applied pressure reduces the spatial separation of silver nanoparticles on the conductive fiber surface, enhancing the probability of electron tunneling. This enables conductive particles to overcome the potential barrier and generate electron movement [40]. The increased number of conductive pathways between fibers reduces contact resistance *R*_e_. During this stage, the simultaneous decrease in both *R*_c1_ and *R*_e_ renders the total resistance highly sensitive to minute pressure changes, significantly improving the sensitivity of EMHFS in the low-pressure range. As the external pressure further increases, the papillary microstructures are compressed to their limit, at which point *R*_c1_ reaches its minimum. Meanwhile, the conductive particles on the fiber electrode surface reach the percolation threshold, causing *R*_e_ to stabilize and no longer change with increasing pressure. However, the flat layer begins to deform under pressure, increasing the number of conductive pathways in the internal network, thereby gradually decreasing *R*_c2_. When the number of pathways saturates, *R*_c2_ is minimized, and the pressure detection upper limit is reached. Upon pressure release, the elastic properties of PDMS allow the piezoresistive layer to quickly return to its initial state, ensuring a reliable response to resistance changes. The pressure response process of EMHFS is further quantified by the sensitivity curve (Fig. 4b). As a key performance parameter for evaluating pressure sensors, sensitivity can be defined as [20]:

7 $$S = \frac{{\delta \left( {\frac{\Delta R}{{R_{0} }}} \right)}}{\delta P}$$

where *P*, *R*_*0*_, and ∆*R* represent the applied pressure, the initial resistance of the sensor, and the change in resistance under applied pressure, respectively. Based on the response process, three linear intervals were identified: S_1_ = 28.72% kPa^−1^ (< 1.47 kPa), S_2_ = 6.37% kPa^−1^ (1.47–14.35 kPa), and S_3_ = 0.23% kPa^−1^ (14.35–105 kPa). The EMHFS demonstrates varying sensitivities across different pressure ranges, corresponding to the three stages of the analysis above.

The high sensitivity of EMHFS stems from the design of its microstructures. When subjected to pressure, surfaces with microstructures exhibit greater mechanical deformation, increasing the contact area and thereby enhancing the sensor's sensitivity. Before delving into the role of microstructure, the influence of CNTs content on sensor performance was first investigated. To identify the optimal CNTs content, the piezoresistive layer was fabricated as a flat-structured film. As the CNTs content increased, the number of conductive pathways within the film grew, progressively enhancing performance (Fig. S22). However, excessive CNTs adversely affected the rheological properties of the PDMS/CNTs composite ink, preventing the formation of microstructures within the mold. Therefore, a 3 wt% CNTs was adopted for subsequent testing. Subsequently, cylindrical and pyramidal microstructures were designed using the same templating method to highlight the sensing characteristics and advantages of the papillary microstructure. To control variables, all microstructures were fabricated using the laser etching method (1064 nm pulse laser) on copper blocks. The laser power was set to 15 W for cylindrical structures, 18 W for pyramidal structures, and 20 W for papillary structures to achieve the desired aspect ratios. These structures maintained identical dimensions (1.5 cm × 1.5 cm) and thickness (≈1 mm) (Fig. S19). Each structural unit also featured consistent dimensions (*d* = 300 μm) and spacing (250 μm) (Fig. S20). Figure S21 displays the morphology and elemental distribution of single papillary structure, demonstrating sample uniformity. Then, finite element analysis (FEA) and modeling were used to analyze the compressive deformation behavior of different microstructures under the same strain (Fig. 4c). Combined with the corresponding pressure variation curves of contact area (Fig. S23), it can be observed that due to the axisymmetric nature of the cylindrical structure, each protrusion undergoes uniform deformation. Consequently, the variation in contact area is minimal, with no distinct stress concentration points. As the strain increases, the deformation of the cylindrical structure consistently concentrates on the circular cross section. This uniform deformation pattern makes it suitable only for scenarios requiring stable contact. Stress in both pyramid and papillary structures concentrates at the tips, but pyramidal structures exhibit larger deformations under the same strain, significantly increasing the contact area in the low-pressure range [41]. However, as strain increases, pyramid deformation rapidly approaches its limit, causing contact area to plateau. Thus, pyramidal structures are only suitable for low-pressure detection scenarios. The gradient deformation of the papillary structure provides both stability and a response to a broader pressure range. Sensitivity curves for different microstructures were further analyzed (Fig. S24). The flat layer, serving as the control group, exhibited the lowest sensitivity and pressure detection range. The stable deformation of the cylindrical structure results in lower sensitivity. Although the pyramidal structure exhibits higher sensitivity, it saturates easily under smaller deformations and loading forces. Clearly, the papillary microstructure exhibited the highest sensitivity and the widest linear range. On the one hand, the rapid increase in contact area at the tips in the low-pressure range gave the papillary microstructure superior sensitivity compared to the pyramidal structure. On the other hand, the external cylindrical structure provided a higher yield strength, enabling continuous structural deformation and widening the sensor's working pressure range. Analysis of the sensing characteristics across different microstructures reveals that the papillary microstructure not only achieves high sensitivity but also accommodates a wider range of pressure detection. Furthermore, the sensitivity remains nearly stable across varying humidity conditions (Fig. S25), indicating the EMHFS's potential for continuous operation in high-humidity environments.

In addition to sensitivity and detection limits, response time is another key parameter for evaluating sensor performance. As shown in Fig. 4d, the EMHFS responds rapidly to pressure loading and unloading, with stable resistance change during the pressure maintenance phase. At 10 kPa pressure, the EMHFS exhibits a response time of 57 ms during loading and a recovery time of 42 ms during unloading, approaching the response time of human tactile sensation (30–50 ms) [42]. Interestingly, even at 80% RH, the EMHFS maintained high response and recovery speeds (Fig. S26). By sequentially applying pressures of 0.18, 0.75, 2.26, 7.23, 13.46, and 23.75 kPa, the EMHFS exhibited corresponding increases in resistance change, demonstrating its high sensitivity to pressure variations (Fig. 4e). Furthermore, the response of EMHFS was stable and consistent during each pressure loading cycle, indicating excellent repeatability. The long-term stability of EMHFS at 1.5 kPa over 13,000 load–unload cycles showed minimal change in resistance (initial change rate: 39.5%, final change rate: 40.3%), demonstrating the EMHFS's outstanding durability (Fig. 4f). Comparing the EMHFS with other reported pressure sensors, it exhibits superior performance in terms of sensitivity, pressure detection range, response/recovery time, and cycling stability (Fig. 4g, Table S3).

### EMHFS for Weak Physiological Signal Monitoring and Gesture-Controlled Robotic Hand

**Fig. 5:**
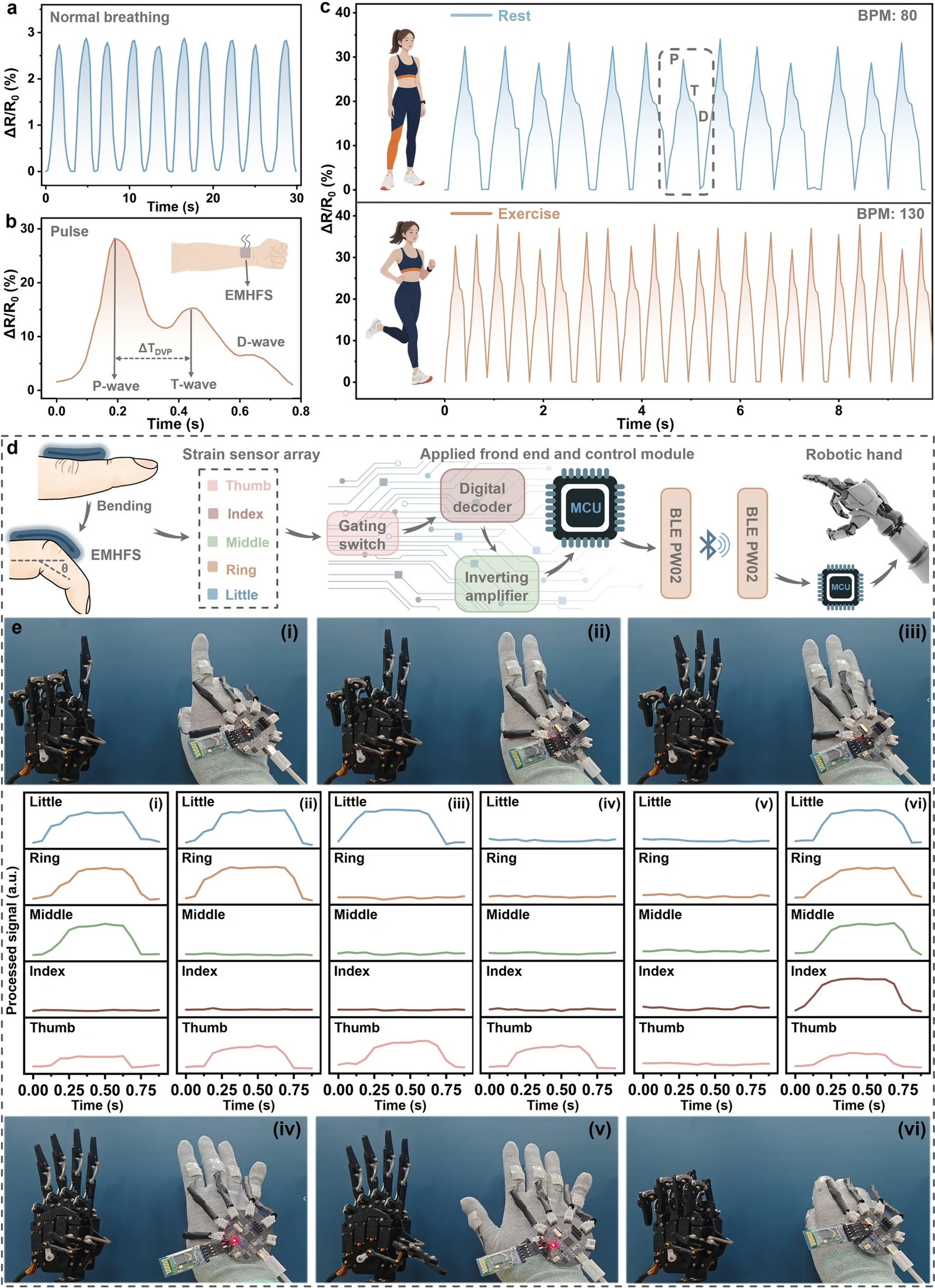
EMHFS for weak physiological signal monitoring and gesture-controlled robotic hand. Resistive response of EMHFS to **a** breathing and **b** pulse signals. **c** Resistance perception signals of EMHFS during volunteer rest and exercise states. **d** Schematic diagram of various modules in the gesture-controlled robotic hand system. **e** Robotic hand mimics the gesture variations through a microcontroller and displays the resistive response of five channels under different gestures

The multifunctional hybrid flexible sensor developed in this study, featuring a fully biomimetic layered structure of eggshell, simultaneously possesses both contact and noncontact sensing capabilities. It can switch sensing modes as required, demonstrating significant application advantages in multimodal HCI. In the contact sensing mode, the EMHFS exhibits excellent detection ability and outstanding operational stability, achieving a wide-range response from low to high pressures. This characteristic, analogous to the eggshell's internal resistance to gradient pressure shocks, provides comprehensive advantages for monitoring weak physiological signals (such as breathing and pulse), gesture-controlled robotic hands, and UAV control systems. The EMHFS was placed beneath the nose of a volunteer for real-time respiratory monitoring. During calm breathing, the generated airflow pressure is less than 0.1 kPa. The resistance change curve in Fig. 5a exhibits periodic, stable fluctuations, indicating that EMHFS can reliably capture pressure signals from respiratory airflow. Pulse signals serve as a critical indicator for enabling early disease prevention. As shown in Fig. 5b, when worn on a volunteer's wrist, the EMHFS captures a pulse signal with distinct waveform characteristics, displaying typical pulse wave components: the percussion wave (P-wave), tidal wave (T-wave), and diastolic wave (D-wave), corresponding to the early systolic pressure peak, late systolic pressure peak, and diastolic pressure, respectively. Changes in these waveforms enable effective assessment of human health status for early prevention of cardiovascular and cerebrovascular diseases. Additionally, we simulated scenarios of wearing the EMHFS on the human body in both resting and exercise states to evaluate its stability under perspiration conditions (Fig. 5c). Results indicate that the EMHFS equipped with directional moisture-wicking functionality remained largely unaffected, retaining clear pulse characteristic waves. Calculations revealed a resting heart rate of 80 beats per minute (bpm) and an exercise heart rate reaching 130 bpm. The distinct heart rate response under different conditions indicates that the EMHFS is adaptable to various physiological states, highlighting its significant application value in wearable health monitoring. Typically, finger bending generates a wide range of pressures (0.1–100 kPa). Therefore, the EMHFS was secured at the finger joint to test its dynamic pressure detection capability. When the finger bent from 0° to 120°, the EMHFS exhibited a stepwise resistance response (Fig. S27). After 20 repetitions of bending at 90°, the resistance changes remained stable and consistent (Fig. S28). Furthermore, we constructed a strain sensing array by attaching EMHFS as sensing units to the joints of the thumb, index, middle, ring, and little fingers. The HCI system based on this array achieved precise control of a robotic hand by tracking finger movements. The system architecture is illustrated in Fig. 5d; when the volunteer performed different gestures, the five channels generated distinct signals. The control module then converts these resistive changes into transmissible electrical signals, which are wirelessly transmitted via a Bluetooth module to drive the robotic hand in corresponding motions. Six action demonstrations showcase the synchronization between human and robotic hand actions (Fig. 5e(i–vi)). The system accurately captures finger motions and controls the robotic hand to replicate them in real time (Movie S1).

### EMHFS-Based Wearable UAV Control System for 3-DoF Stable Control

**Fig. 6:**
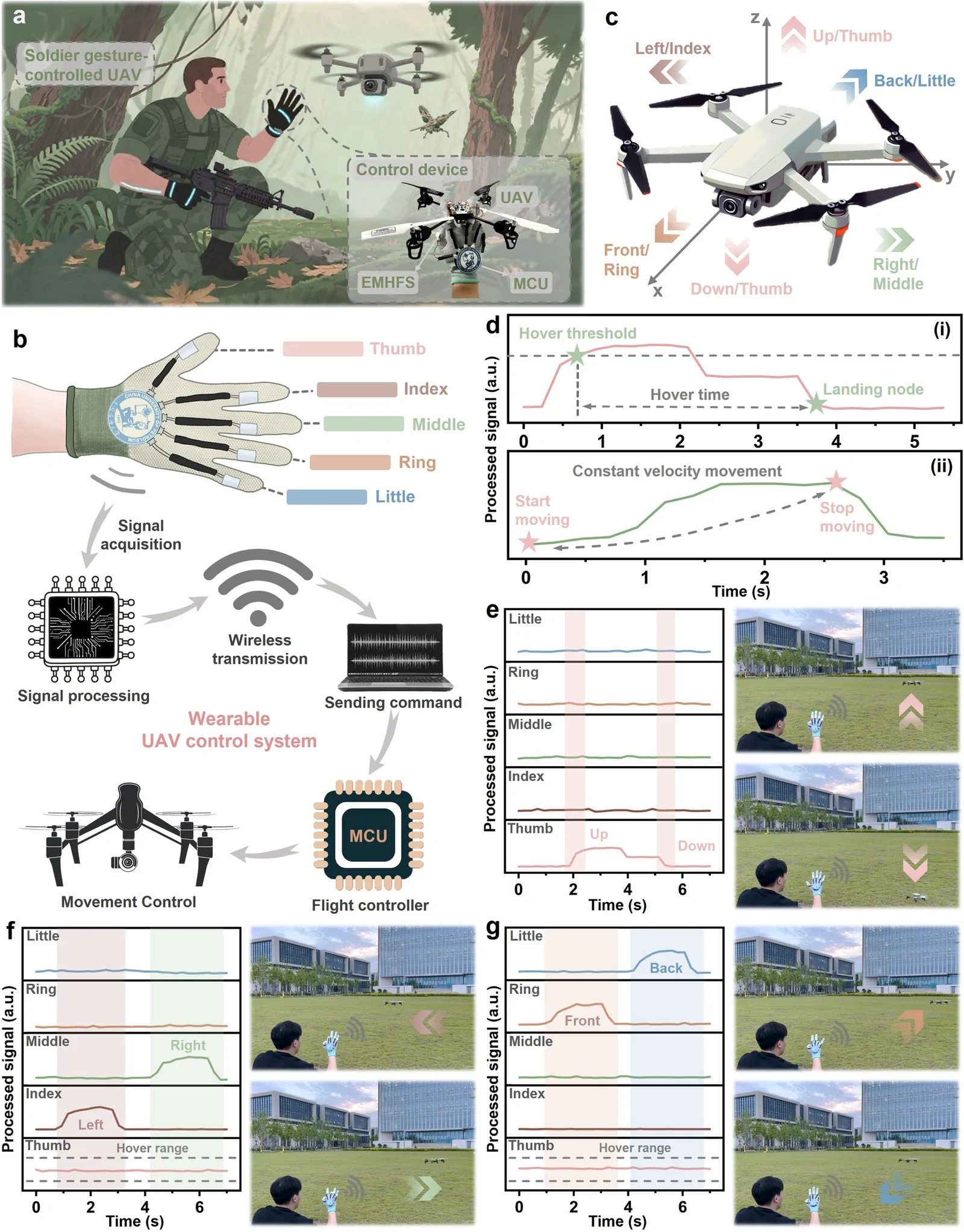
Implementation of the EMHFS-based wearable UAV control system for 3-DoF stable control. **a** Schematic illustration of a soldier controlling UAV via hand gestures in a silent jungle environment, with an accompanying photograph of the control device. **b** Schematic diagram depicts various modules and operational processes of the EMHFS-based wearable UAV control system. **c** Five channels in the system correspond to the 3-DoF for UAV control. **d** Control rules for manipulating UAV movement via (i) thumb and (ii) other finger channels. EMHFS acts as a flight controller to control the UAV's movement in **e** vertical, **f** lateral, and **g** longitudinal directions, along with the corresponding command signals

Given the exceptional contact sensing capabilities of EMHFS, we further explored its potential applications in more complex HCI scenarios. In special forces missions, soldiers often need to maintain a low profile and high mobility in complex environments. In this study, a UAV control system based on the EMHFS sensor array was developed. Unlike the traditional remote control that requires constant observation and operation, soldiers in a jungle environment can use covert gestures to silently control the UAV for reconnaissance, without exposing their position (Fig. 6a). The UAV control system workflow consists of five core steps: signal acquisition, signal processing, wireless transmission, command dispatch, and motion control (Fig. 6b). Initially, the EMHFS sensor array is integrated into the finger joints. Finger bending alters resistance changes, converting mechanical strain from gestures into electrical signals, which completed the initial signal acquisition. The acquired signals are then transmitted to the signal processing module, where the signals are amplified, filtered, and converted into digital signals that the system can recognize. The processed signals are wirelessly transmitted to the UAV control end, where they are decoded into control instructions for the UAV's flight controller. Upon receiving the control instructions, the flight controller adjusts the motor speeds to achieve the corresponding UAV movements.

Figure 6c illustrates the spatial mapping for finger-controlled UAV movement directions, corresponding to vertical motion controlled by the thumb and horizontal motion controlled by other fingers. Leveraging the EMHFS's high sensitivity across a wide pressure range, control rules for maneuvering the UAV were defined (Fig. 6d). In the vertical direction (up/down), signals generated by thumb bending are received by the control unit, causing the UAV to ascend. When the thumb's bending signal reaches a threshold, the UAV enters a hover state. In this state, the UAV's altitude is locked, and further thumb bending no longer controls height. The hover duration is determined by how long the signal remains in the threshold range. When the thumb returns to its initial angle, the UAV exits hover mode and begins the landing sequence (Fig. 6d(i)). In the horizontal direction, each finger follows identical control rules: when a finger bends, the UAV moves in the corresponding direction at a constant speed. Motion ceases only when the finger returns to its initial angle (Fig. 6d(ii)). Notably, the complex UAV maneuvers result from the coordinated control of the thumb and other fingers. In actual use, the thumb first controls the UAV to ascend to a designated height (Fig. 6e). When the thumb's bending signal remains within the hovering range, the index, middle, ring, and little fingers control the UAV to move left, right, forward, and backward, respectively (Fig. 6f, g). Finally, the thumb controls the UAV to complete the landing. Overall, the wearable UAV control system developed in this study achieves stable three degree of freedom (3-DoF) motion control through precise mapping of finger movements to the three-dimensional (3D) spatial motion (Movie S2).

### EMHFS for Password and Gesture Unlocking on Touchless Screens

**Fig. 7:**
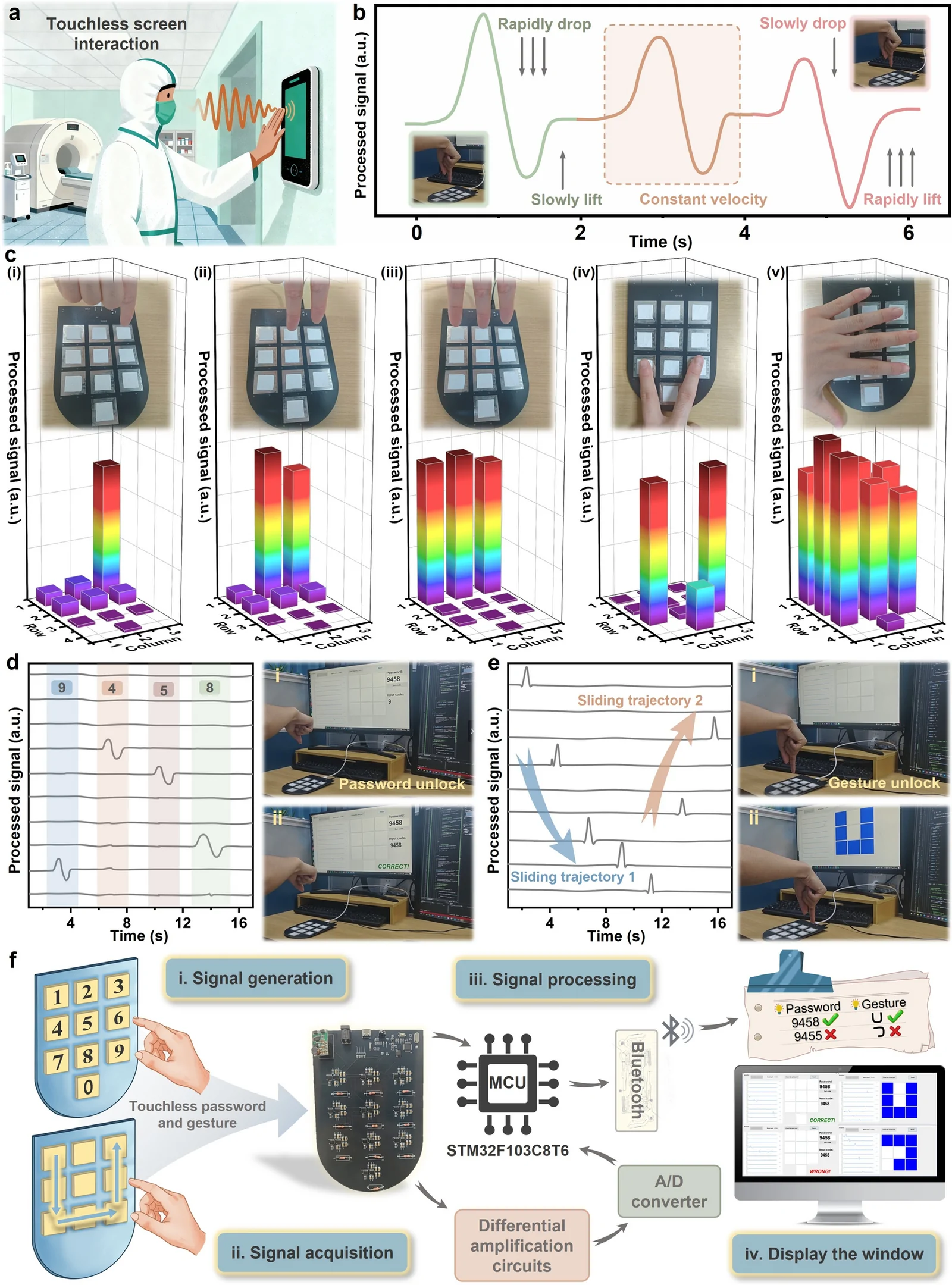
Application demonstration of EMHFS for password and gesture unlocking on touchless screens. **a** Schematic diagram of integrating EMHFS into touchless screens for contactless operation scenarios in hospital public devices. **b** Response signals generated when fingers rise and fall at different vertical speeds. **c** Response signals corresponding to each unit depicted by the 3D diagram as the hand moves across the EMHFS unit array in different postures. **d** Response signal from the ten units when the preset password ‘‘9–4–5–8'' is entered sequentially using the finger; the accompanying diagram demonstrates the interface for unlocking via password entry in the touchless operation mode based on EMHFS. **e** Response signal from ten units when sliding finger in a preset ‘‘U'' shape gesture. The attached diagram demonstrates the touchless operation mode based on EMHFS, showing the interface unlocked via gesture input. **f** Working process and unlocking mechanism diagram of the touchless screen control system

Although contact-based screen sensors are widely used in healthcare, their limitations become increasingly apparent with the continuous advancement of medical technology and rising healthcare demands. In pathogen-dense environments like hospitals, contact screens pose a risk of cross-contamination due to direct contact with patient's bodies. Given the exceptional triboelectric performance of EMHFS in noncontact sensing modes, we developed an intelligent touchless screen control system based on EMHFS. This system successfully demonstrated applications such as touchless screen passwords and gesture unlocking, offering an infection-free alternative in hospital public equipment settings (Fig. 7a).

In this study, functional circuits for password and gesture unlocking were first designed, and the circuit operation principle was explained (Fig. S30). Through rational structural design and wiring, the relevant electronic components and modules were integrated on the back of the PCB, while the front comprised an array of ten metal plate electrodes (Fig. S31). The physical image of the PCB is shown in Fig. S32. Ten EMHFS sensing units (each measuring 1.5 cm × 1.5 cm) were fixed onto the array electrodes to form the front-end sensing device. The equivalent circuit of the sensor array is shown in Fig. S33. To minimize crosstalk between adjacent sensing units, their dimensions were made smaller than the size of the metal plate electrodes, and the distance between the signal paths was increased. This effectively minimized crosstalk between adjacent sensor units. Figure 7b displays the response signals generated by finger movements at different vertical speeds during one cycle, with the inset illustrating the motion trend. When the finger drops rapidly, a narrow, high electrical signal is produced in real time. As the finger slowly lifts, a relatively wide and low electrical signal is generated. Similarly, when the finger drops slowly followed by a rapid lift, distinguishable electrical signal features appear. When the finger moves at a constant speed, a symmetrical waveform is produced relative to the center point. The differences in peak values and slopes of the signals generated at different speeds suggest that the sensing device can accurately identify the speed characteristics of finger movements, providing a foundation for subsequent gesture and password unlocking applications. The electrical signal response from single EMHFS sensing unit is insufficient to distinguish complex gestures. Therefore, further testing examined the response signals from each unit when the hand moved across the EMHFS array in different poses. Figure 7c presents a 3D signal plot clearly showing the consistency of the electrical signal responses with the locations of the involved sensor units. The significant differences in signal intensity and distribution corresponding to different postures indicate that the sensing device can distinguish the spatial orientation of hand movements, supporting the diversity of gesture recognition. Furthermore, a real-time intelligent screen password and gesture unlocking interface was designed. The initial interface for touchless screen password unlocking is shown in Fig. S34, where the ten digits from 0 to 9 correspond to ten sensing units of the EMHFS. When fingers sequentially input the digits ‘‘9–4–5–8,'' each digit generates a corresponding electrical signal response from the specific sensor unit (Fig. 7d). Since the preset password is 9458, upon receiving the input signals, the screen displays a password correct prompt, successfully unlocking the device via touchless operation (Fig. 7d(i, ii) and Movie S3). Notably, the sensor unit for the digit “0'' was eliminated to enable touchless screen gesture unlocking (Fig. S35). When the finger traces a ‘‘U'' shaped gesture, corresponding sensor units along the sliding trajectory generate distinct signal responses (Fig. 7e and Movie S4). Additionally, an ‘‘L'' shaped gesture application example was demonstrated, validating the system's real-time dynamic noncontact sensing capability (Movie S5).

Figure 7f details the workflow and unlocking mechanism of the touchless screen control system. The system operates in four stages: signal generation, signal acquisition, signal processing, and display window. When the user performs a touchless password or gesture operation, EMHFS sensors are triggered. The MCU in the sensing device acquires the raw signal, processes it through differential amplifier and analog-to-digital conversion circuits before being transmitted wirelessly via Bluetooth to the receiving end. The terminal interface verifies the password or gesture, enabling touchless screen unlocking. These demonstrations showcase the EMHFS's applications in touchless screen password and gesture unlocking, highlighting its noncontact, high precision, and multimodal recognition advantages. This provides a viable solution for contactless interaction with public equipment in hospitals.

## Conclusion

This study presents a fully biomimetic multifunctional flexible sensor inspired by the natural layered structure of eggshells (EMHFS). By mimicking the eggshell's cuticle layer, spongy layer, papillary layer, and inner–outer shell membranes, EMHFS achieves functional integration across four layers. The cuticle layer-inspired triboelectric layer comprises electrospun PVDF/MXene/BaTiO_3_ nanofibers and utilizes the triboelectric effect and electrostatic induction to achieve excellent noncontact sensing performance, with a maximum detection range of up to 2 m. The spongy and papillary-inspired PDMS/CNTs piezoresistive layer, fabricated via a template method, achieves high sensitivity (28.72% kPa^−1^), a wide pressure detection range (up to 105 kPa), fast response/recovery times (57/42 ms), and outstanding durability (stable performance after 13,000 loading–unloading cycles). The inner–outer shell membrane-inspired PAN/TPU nanofiber hydrophilic–hydrophobic bilayer membrane exhibits good directional moisture absorption, breathability, and antibacterial properties (inhibition rates of 92.9% against *E. coli* and 91.2% against *S. aureus*). The fully biomimetic design maximizes the complementary advantages of each layer, enabling seamless switching between different sensing modes. This novel design facilitates diverse HCI applications, including real-time gesture-controlled robotic hands via a strain sensing array, wearable UAV control systems for stable 3-DoF control, and touchless screen unlocking for password and gesture recognition. It also demonstrates significant advantages in monitoring weak physiological signals like breathing and pulse, even in high-humidity environments. Overall, this innovative biomimetic approach overcomes the limitations of traditional sensors with single mode operation, opening new possibilities for next-generation wearable electronics, smart structures, and multimodal HCI systems.

Despite the significant advantages of EMHFS, this study still has certain limitations. First, the current preparation process relies on manual stacking of layers, which limits large-scale production efficiency. Second, the sensor's performance in extreme temperature environments has not been fully verified. In future work, we will develop a continuous roll-to-roll manufacturing process to improve production scalability and systematically investigate the sensor's environmental stability under extreme temperatures. Additionally, we plan to integrate wireless power supply modules to extend the application scope of the sensor in long-term wearable scenarios.

## Supplementary Information

Below is the link to the electronic supplementary material.

Supplementary file1 (MP4 6996 KB)

Supplementary file2 (MP4 7000 KB)

Supplementary file3 (MP4 3377 KB)

Supplementary file4 (MP4 2606 KB)

Supplementary file5 (MP4 1141 KB)

Supplementary file6 (DOCX 15380 KB)
