# Supplementary material for: A Fully Biomimetic Flexible Sensor Inspired by the Natural Layered Structure of Eggshells for Multimodal Human–Computer Interaction
Source: Nanomicro Lett. 2026 Feb 9;18:244. doi: 10.1007/s40820-026-02101-2 (PMC12886703; doi:10.1007/s40820-026-02101-2)
Supplement: Supplementary file 6 — Supplementary file6 (DOCX 15380 KB) [file 40820_2026_2101_MOESM6_ESM.docx]

Supporting Information for

**A Fully Biomimetic Flexible Sensor Inspired by the Natural Layered Structure of Eggshells for Multimodal Human-Computer Interaction**

Weiwei He^1, 2^, Yanzhen Zhang^1, 2,^ *, Puye Zhang^1, 2^, Yunlong Liu^1, 2^, Guanyang Wu^1, 2^, Boce Xue^1, 2^, Guoqing Hu^1, 2^, Runsheng Li^3^, Chao Zheng^1, 2^, and Dongzhi Zhang^4,^ *

^1^ Shandong Key Laboratory of Design and Manufacturing for High-end Offshore Oil and Gas Equipment, College of Mechanical and Electronic Engineering, China University of Petroleum (East China), Qingdao 266580, P. R. China

^2^ National Engineering Research Center of Marine Geophysical Prospecting and Exploration and Development Equipment, China University of Petroleum (East China), Qingdao 266580, P. R. China

^3^ Aviation Services Research Centre, The Hong Kong Polytechnic University, Hong Kong 999077, P. R. China

^4^ State Key Laboratory of Chemical Safety, College of Control Science and Engineering, China University of Petroleum (East China), Qingdao 266580, P. R. China

* Corresponding authors. E-mail: [zhangyanzhen@upc.edu.cn](mailto:zhangyanzhen@upc.edu.cn) (Yanzhen Zhang); [dzzhang@upc.edu.cn](mailto:dzzhang@upc.edu.cn) (Dongzhi Zhang)

**S1 Experimental Section**

**S1.1 Materials**

Ti_3_C_2_T_x_-MXene was prepared according to the procedure in our previous work [S1]. Lithium fluoride (LiF) and Hydrochloric acid (HCl) were obtained from Sinopharm Chemical Reagent Co., Ltd. Acetone, Dimethylformamide (DMF), Polyvinylidene fluoride (PVDF), Tetrahydrofuran (THF), Polyacrylonitrile (PAN), Thermoplastic polyurethane (TPU), Polyvinyl alcohol (PVA), Citric acid (C_6_H_8_O_7_), Sodium citrate (C_6_H_5_Na_3_O_7_), Sodium dodecyl sulfonate (SDS), Sodium tetraborate decahydrate, and Gelatin were provided by Shanghai Aladdin Biochemical Technology Co., Ltd. Barium titanate (BaTiO_3_,100 nm) and Carbon nanotubes (CNTs), Glycerol, and Sodium chloride (NaCl) were purchased from Macklin Biochemical Technology Co., Ltd. PDMS prepolymer (Sylgard 184A) and curing agent (Sylgard 184B) were supplied by Dow Corning (USA). Deionized water for experiments was self-made. All chemicals were used as received without further purification.

**S1.2 Preparation of** **Triboelectric Layer**

The detailed preparation process of triboelectric layer was shown in Fig. S1. Firstly, MXene (0.03 g) and BaTiO_3_ nanoparticles (0.1 g) were added to a solution of Acetone and DMF (8 g, weight ratio of 7:3), and the mixture was ultrasonicated in an ice bath to obtain the dispersion. Subsequently, 2 g of PVDF particles were dissolved in the dispersion and stir magnetically at 70 ℃ for 2 h to obtain the spinning solution. Finally, a 5 mL aliquot of the spinning solution was loaded into a plastic syringe fitted with a metal needle. Electrospinning was performed at 15 kV high voltage with a feed rate of 0.35 mm/min for 2 h to obtain the PVDF/MXene/BaTiO_3_ nanofiber membrane (triboelectric layer). The spinning environment was maintained at 35 ℃ and 40% RH. The distance between the needle and the collector was set to 20 cm.

**S1.3 Preparation of Piezoresistive Layer**

To prepare the piezoresistive layer (Fig. S2), a laser etching method with a Gaussian energy distribution was employed to etch patterns within the copper block. By adjusting the laser power and geometric shapes in EZCAD, microstructures of various shapes could be obtained. Then, a solution of PDMS (2 g, weight ratio of prepolymer: curing agent = 10:1) with 3 wt.% CNTs was stirred and mixed, and the resulting mixed solution was transferred to a rotary vane vacuum pump (VP-1, Taizhou Zhenkong Pump Co., Ltd.) for vacuum debubbling. Finally, the mixture was poured on copper molds to form piezoresistive layers with distinct microstructures. After solidifying at 80 ℃ oven for 2 h，the cured piezoresistive layers could be easily peeled off.

**S1.4 Preparation of Hydrophilic and Hydrophobic Layers**

Fig. S3 illustrated the preparation details of hydrophilic and hydrophobic layers: 1) PAN particles were dissolved in DMF with 0.2 wt.% SDS to obtain the PAN spinning solution (10 wt.%). Electrospinning was performed at 15 kV high voltage with a feed rate of 0.35 mm/min for 2 h to obtain the PAN nanofiber membrane (hydrophilic layer) with the thickness of about 60 μm. The spinning environment was maintained at 35 ℃ and 40% humidity. The distance between the needle and the collector was set to 15 cm. 2) TPU particles were dissolved in a mixed solvent of THF and DMF to obtain the TPU spinning solution (20 wt.%). Using the same spinning parameters, spinning was continued onto the PAN nanofiber membrane for 1 h to obtain the TPU nanofiber membrane (hydrophobic layer) with the thickness of about 20 μm.

**S1.5 Preparation of Nanofiber Electrodes**

Magnetron sputter-deposited silver was applied to the bottom of the triboelectric layer and the surface of the hydrophilic layer to form nanofiber electrodes for triboelectric performance and piezoresistive layer testing.

**S1.6 Preparation of Temperature-Controlled Adhesive Gel**

Firstly, citric acid (0.08 g) and sodium citrate (0.8 g) were dissolved in an aqueous solution of glycerol (8 g, weight ratio of 1:1). Then, 2 g of gelatin and 0.5 g of NaCl were added to the solution and stir in a 70 ℃ water bath until the mixture was dissolved completely. Mixing 1 g of the pre-prepared PVA solution (15 wt.%) with the resulting solution. While heating and stirring the mixture, borax (0.1 g) was slowly added until the solution transformed into a homogeneous gel. Finally, the adhesive gel was obtained as the temperature cooled (Fig. S4).

**1.7 Preparation of EMHFS**

The triboelectric layer, piezoresistive layer, hydrophilic layer, and hydrophobic layer were stacked sequentially from top to bottom. The adhesive gel was applied around the edges using a fine-bristled soft brush. After trimming the excess material, the EMHFS with dimensions of 1.5 cm×1.5 cm was obtained. Within the EMHFS, the bottom of the triboelectric layer and the top of the hydrophilic layer served as the upper and lower electrodes for the piezoresistive layer, respectively, connected to external devices via conductive copper wires.

**S1.8 Characterization and Measurement**

The morphology and structure were observed using scanning electron microscopy (SEM, EM30-PLUS, COXEM) with energy dispersive spectroscopy (EDS, Oxford, UK) at 10 kV in low vacuum mode. The triboelectric performance of EMHFS was determined by an oscilloscope (Keysight DSOX2012A) and an electrostatic meter (model 6514, Keithley, USA). Notably, the baseline of the voltage signal was calibrated to 0 V for better comparison. The water contact angle was measured using a contact angle measuring instrument (Y-PHb, Changzhou Taylor Instrument Technology Co., Ltd., China). The antimicrobial properties of EMHFS were investigated by bacterial suspension method by inoculating 1 mg of the sample into the bacterial fluids of *S. aureus* and *E. coli*, and incubating them at 37 ℃ temperature. OD_600_ was recorded at different time intervals using an enzyme marker. The pressure sensing performance of EMHFS were evaluated using a self-assembled platform integrating an LCR meter (IM3536, HIOKI, Japan) for electrical measurements, a universal testing machine (UTM4204, Sansheng Technology Co., Ltd., China) for mechanical loading, and a PC host for data acquisition and analysis.

**Supplementary Figures and Tables**


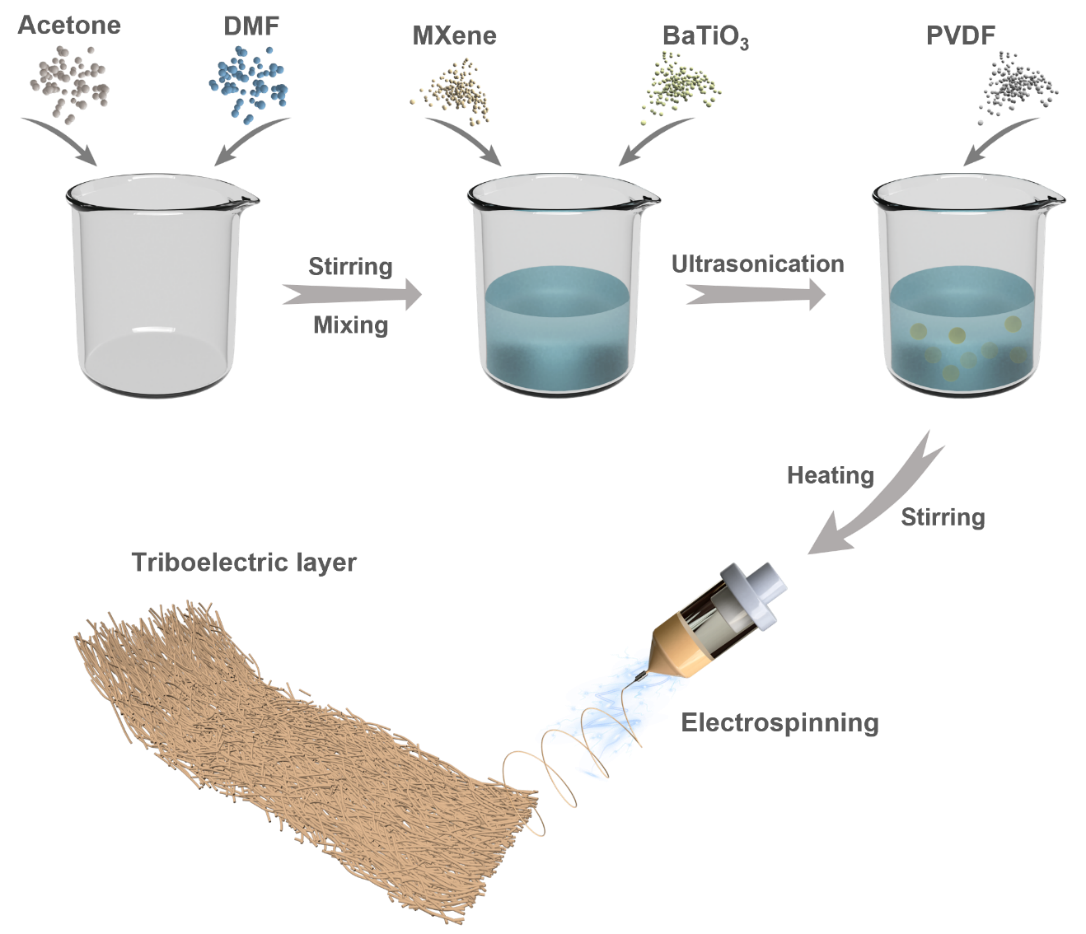


**Fig. S1** Preparation of triboelectric layer


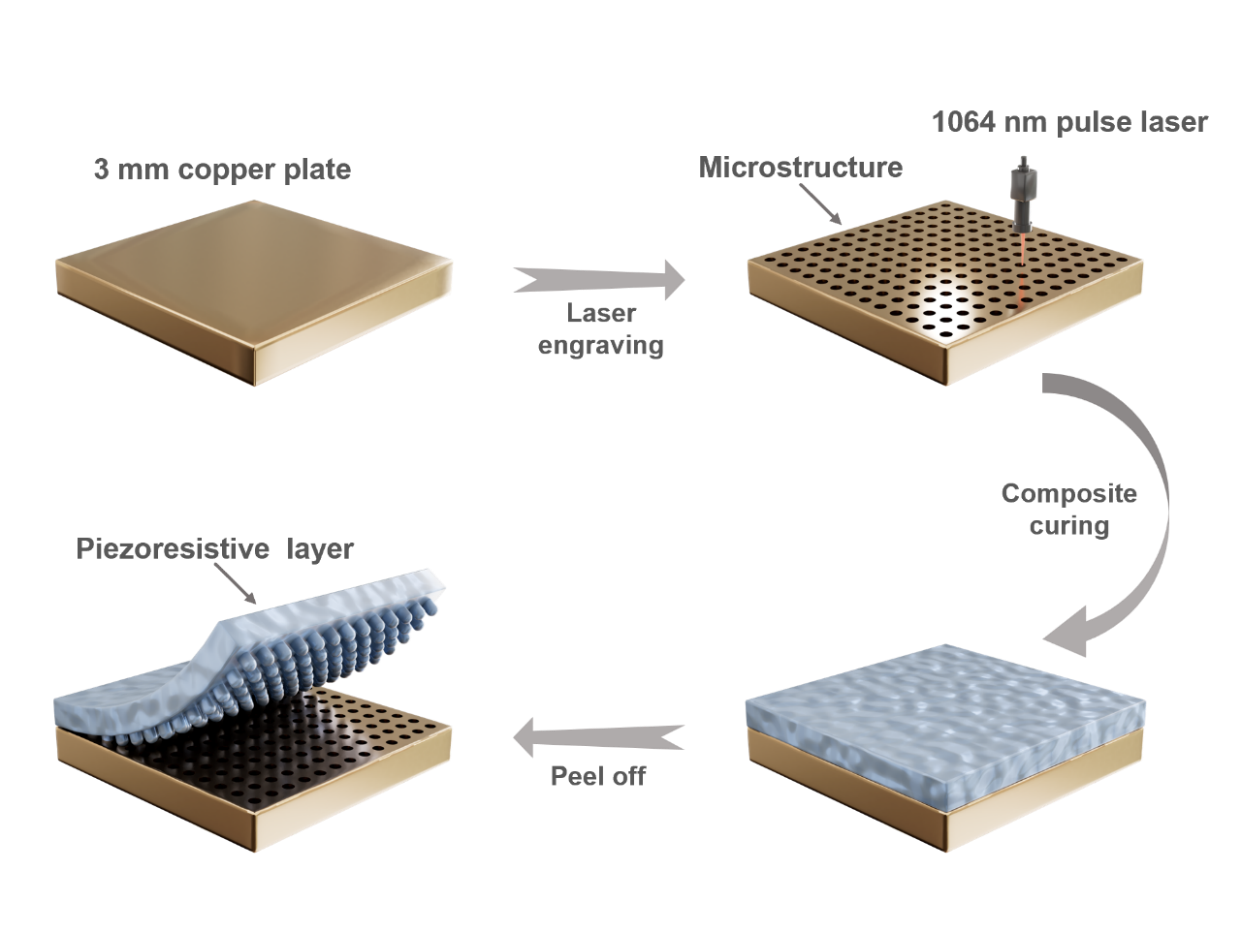


**Fig. S2** Preparation of piezoresistive layer


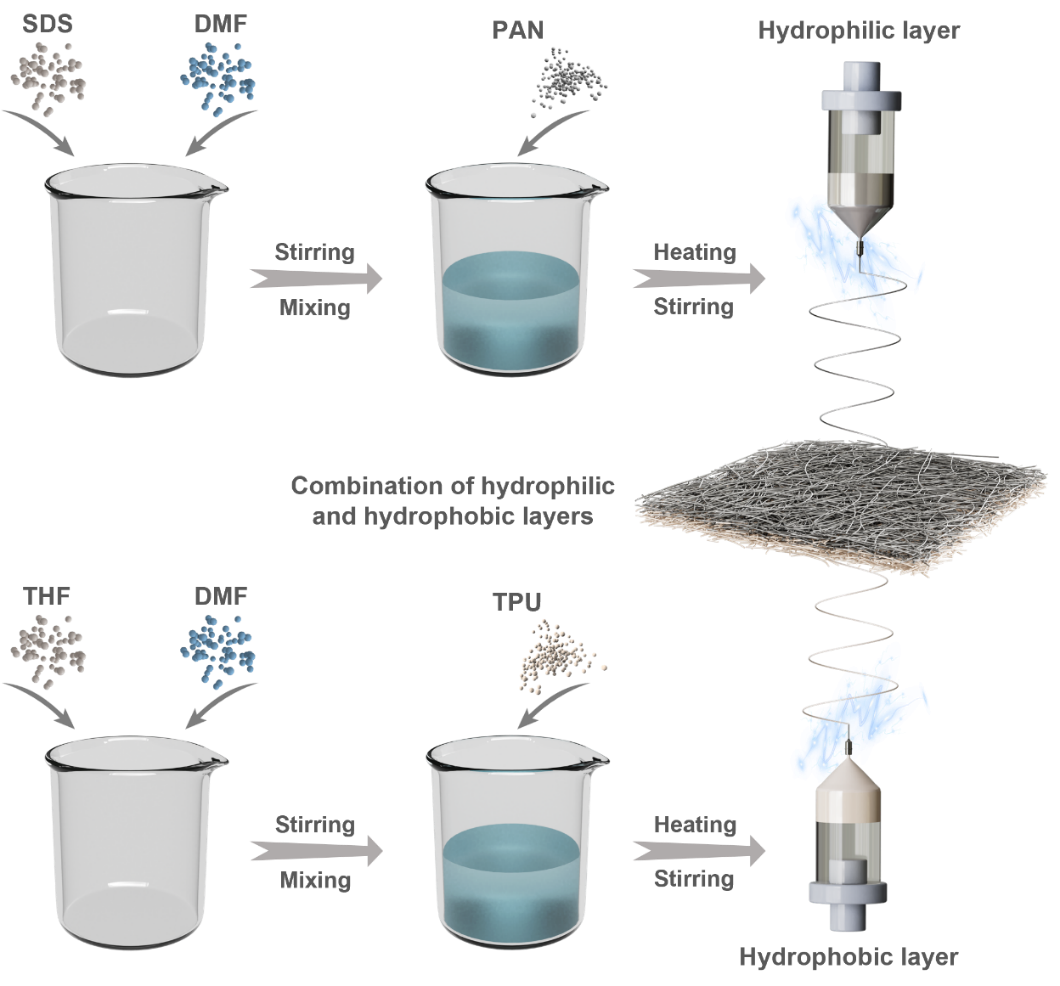


**Fig. S3** Preparation of hydrophilic and hydrophobic layers


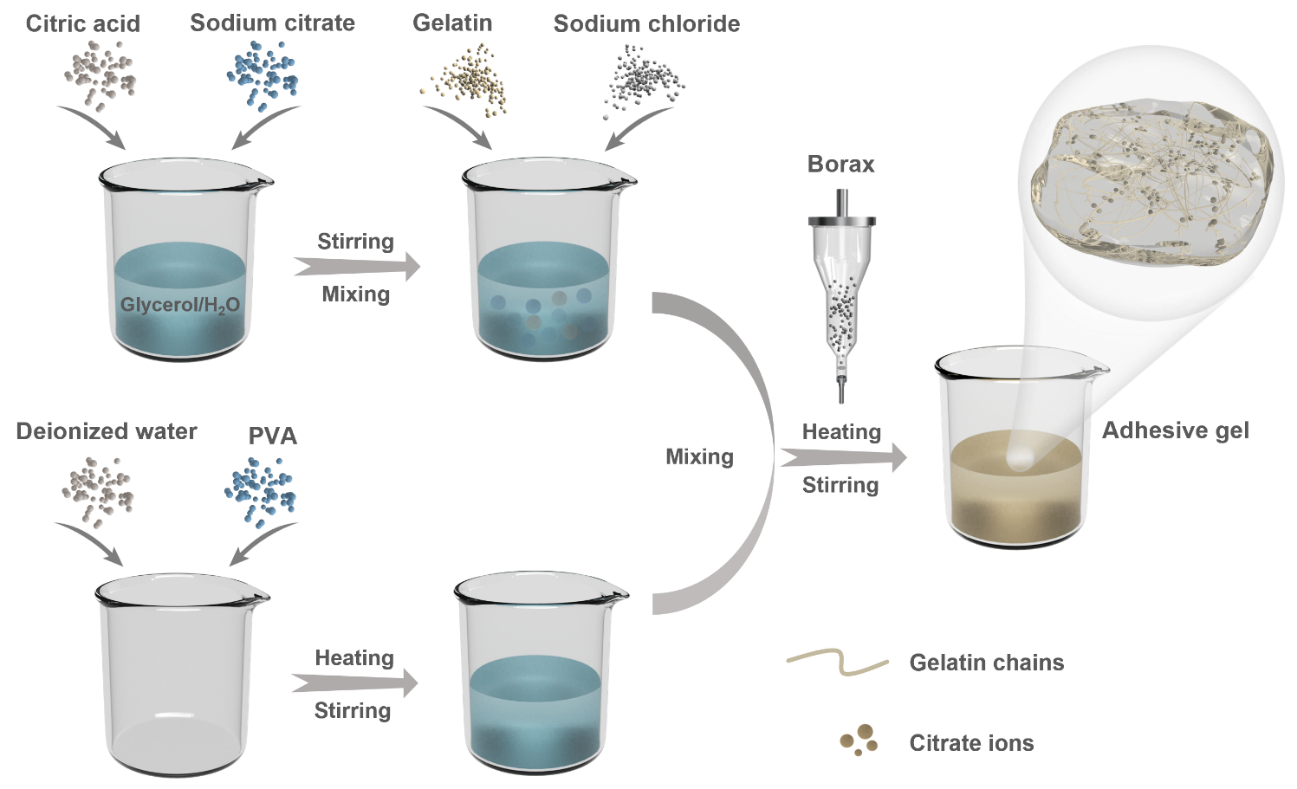


**Fig. S4** Preparation of temperature-controlled adhesive gel


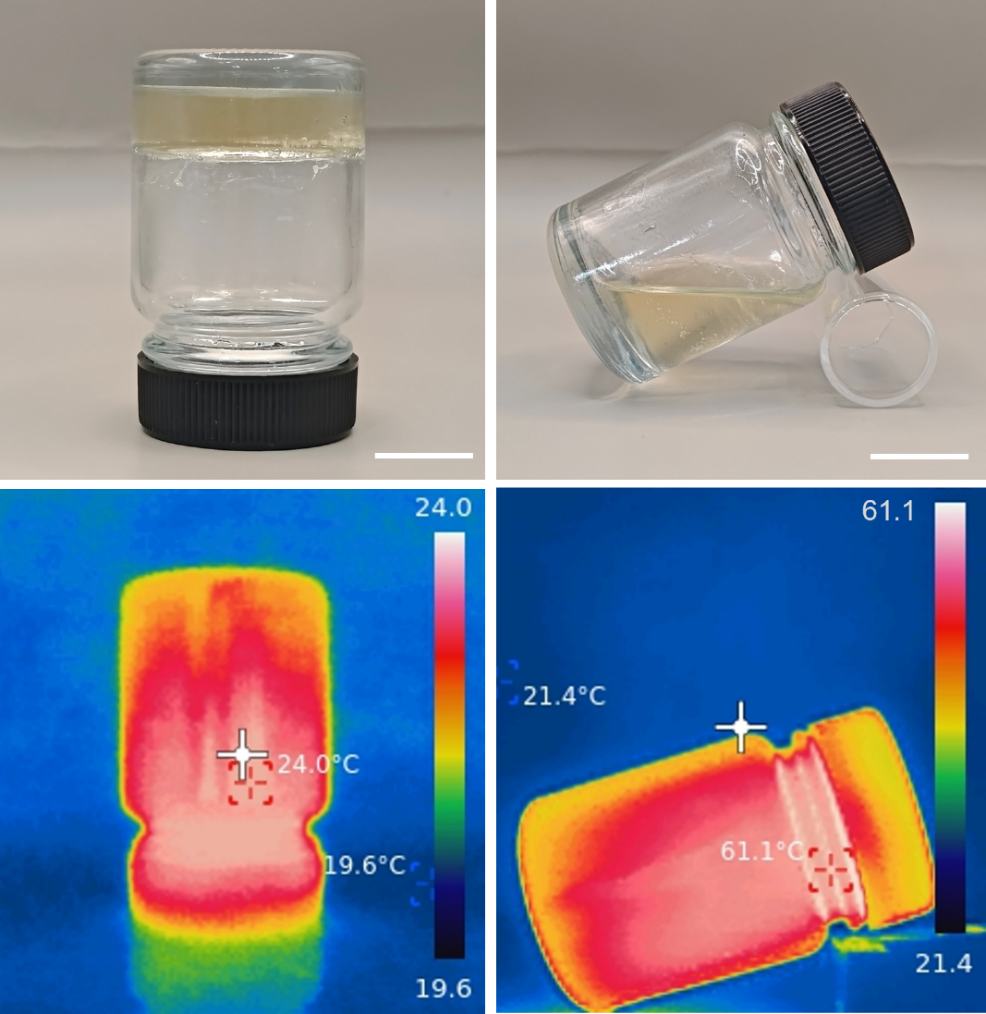


**Fig. S5** Photographs and infrared camera images of adhesive gel at room temperature and high temperature. Scale bars, 1 cm

The EMHFS was encapsulated integrally using a self-made thermally controlled adhesive gel. Specifically, due to the material selection and preparation techniques, the adhesive gel exhibits reversible non-covalent cross-linking. This property allows the gel to exist as a fluid above 40 °C and transform into a solid gel state at room temperature (Fig. S5), at which point it possesses high extensibility. The fluid gel was applied around the edges of each layer of the EMHFS using a fine brush. After stacking the layers, gentle compression is applied. Upon cooling to room temperature, the encapsulation of the EMHFS is complete


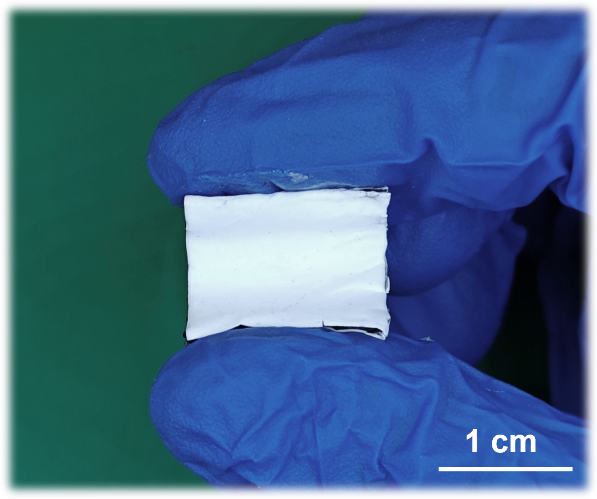


**Fig. S6** Photograph of EMHFS in a bent state


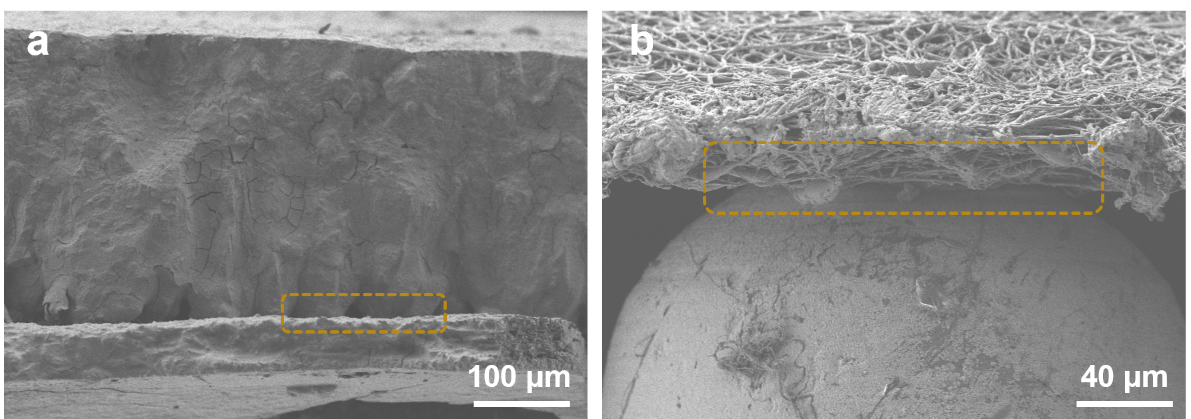


**Fig. S7** Cross-sectional morphologic structure of **a** eggshell and **b** EMHFS


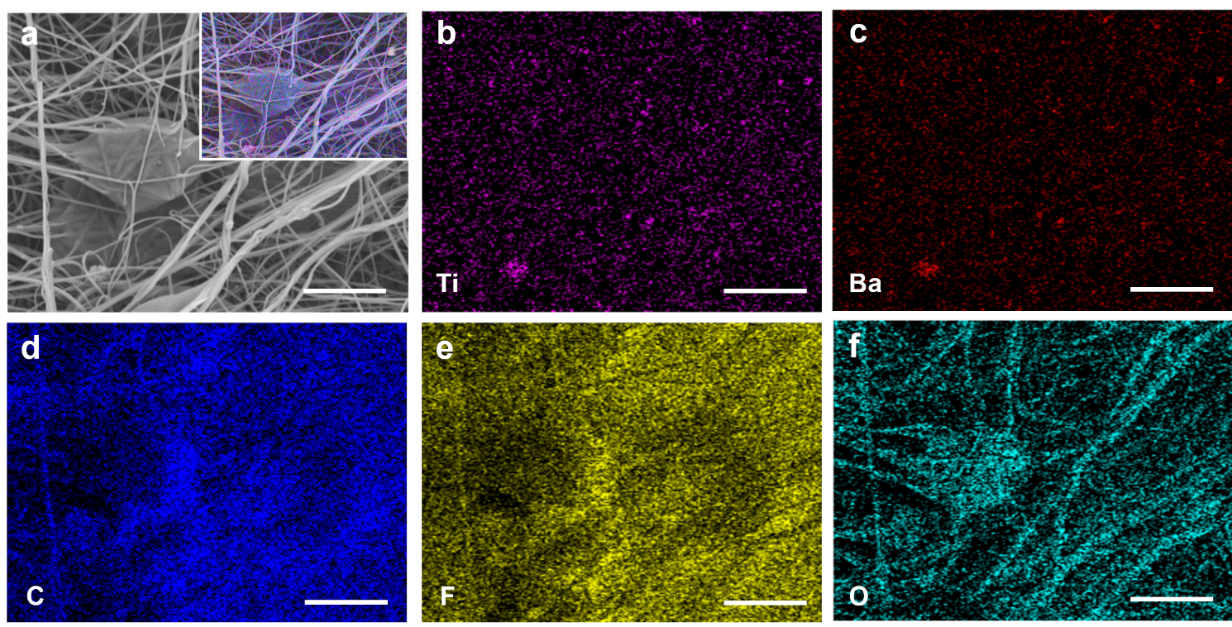


**Fig. S8** EDS mapping of **a** the top of friction layer with element distribution images of **b** Ti, **c** Ba, **d** C, **e** F, and **f** O. Scale bars, 10 μm


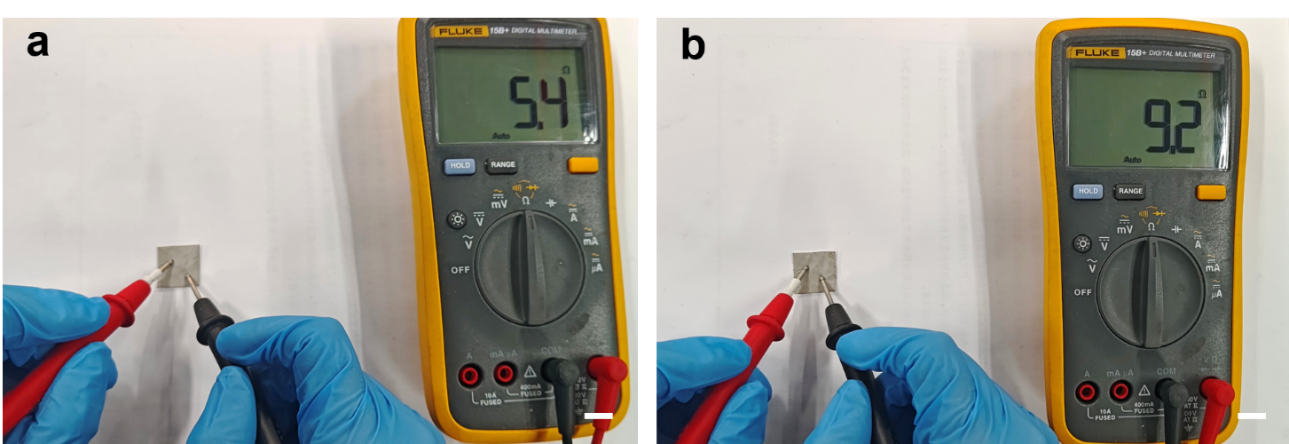


**Fig. S9** Photograph showing the measurement of the highly conductive **a** bottom electrode of the triboelectric layer and **b** surface electrode of the hydrophilic layer. Scale bars, 1 cm
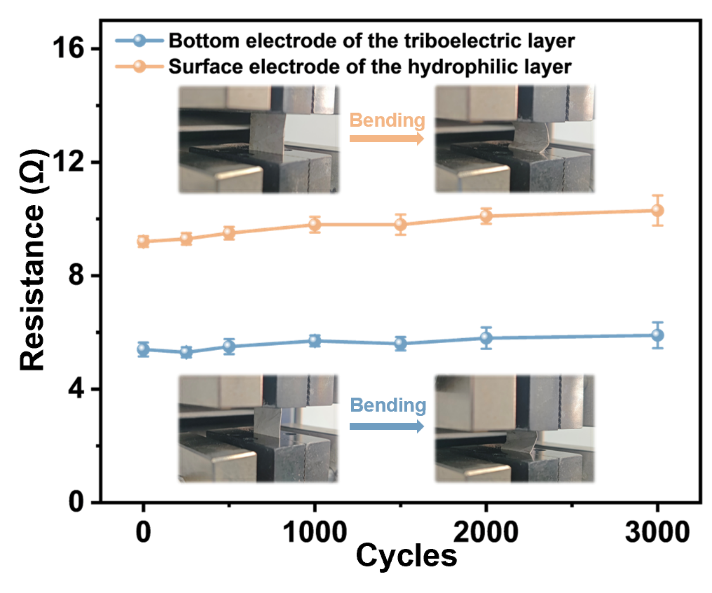


**Fig. S10** Resistance changes of bottom electrode of the triboelectric layer and surface electrode of the hydrophilic layer under different number of bending cycles


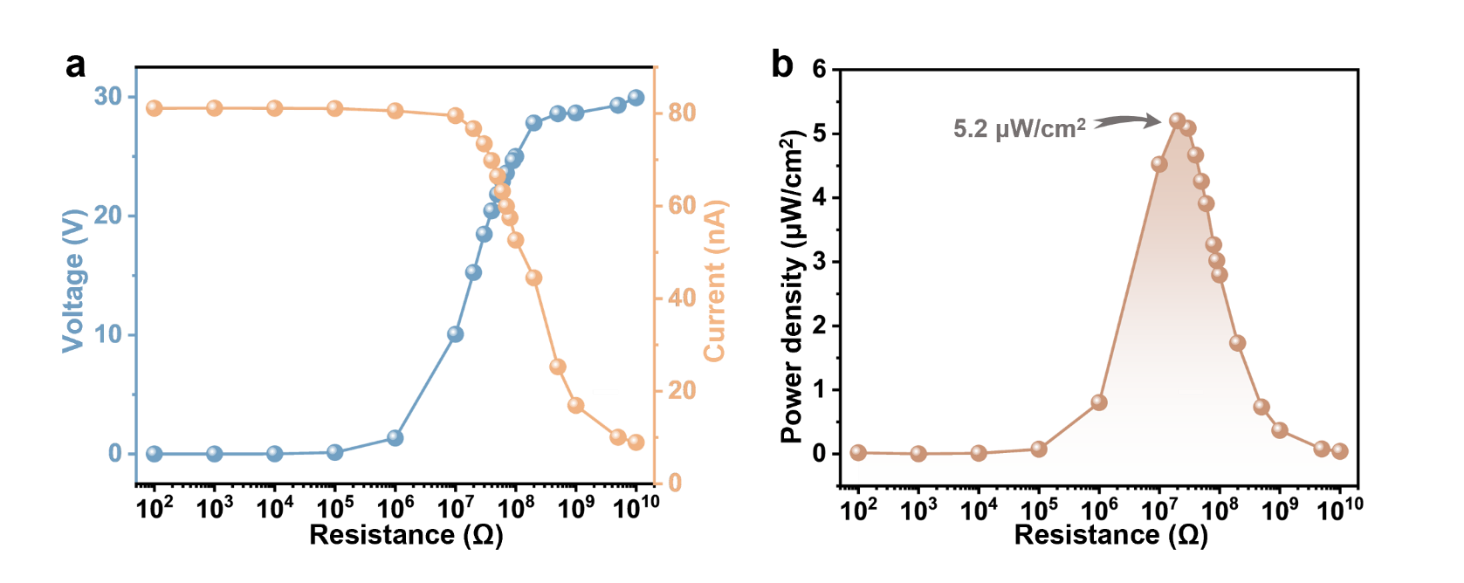


**Fig. S11 a** Output voltage, current, and **b** instantaneous peak power density of the EMHFS under a series of external loads


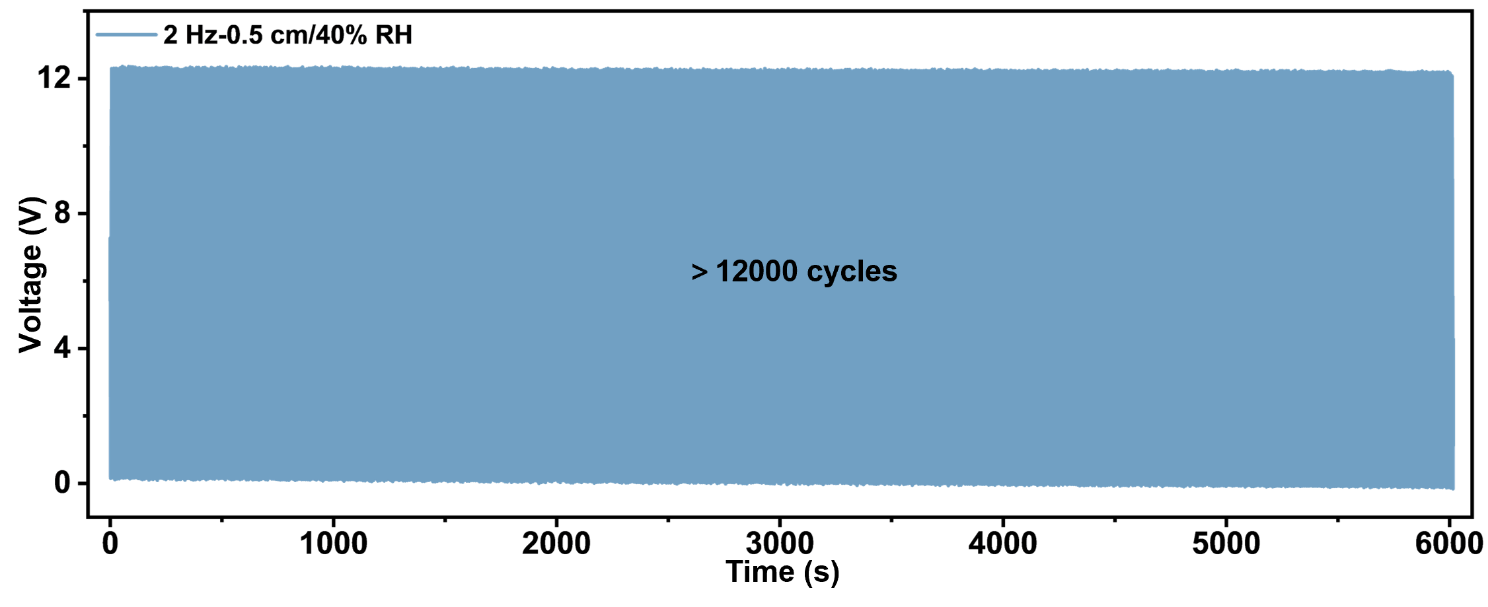


**Fig. S12** The working durability testing of EMHFS


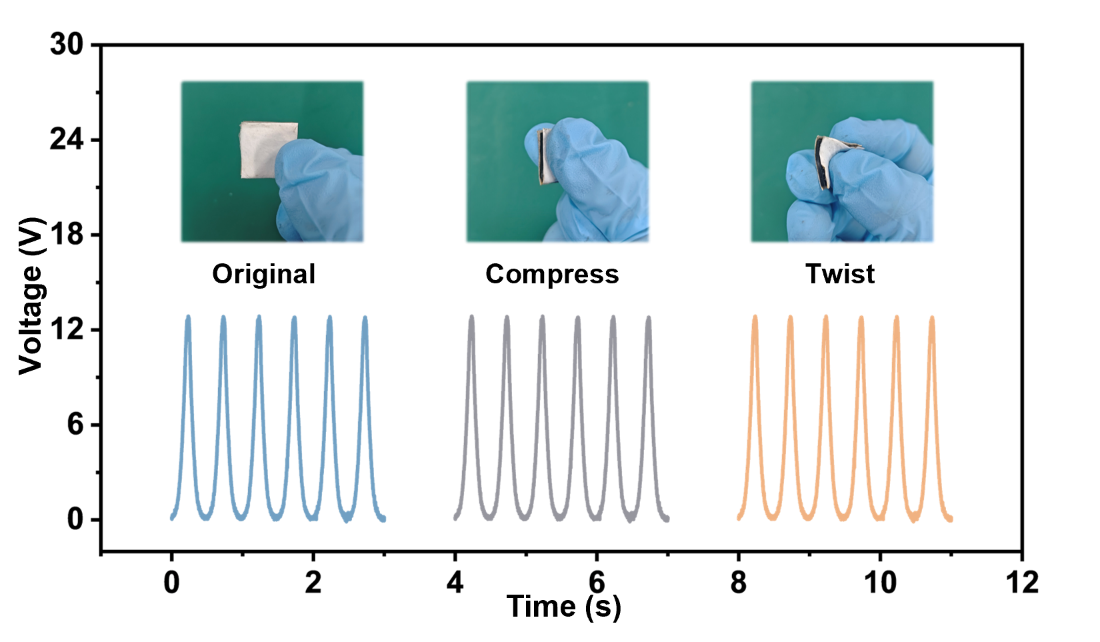


**Fig. S13** Comparison of the output voltage provided by EMHFS in original state versus after compression and torsion


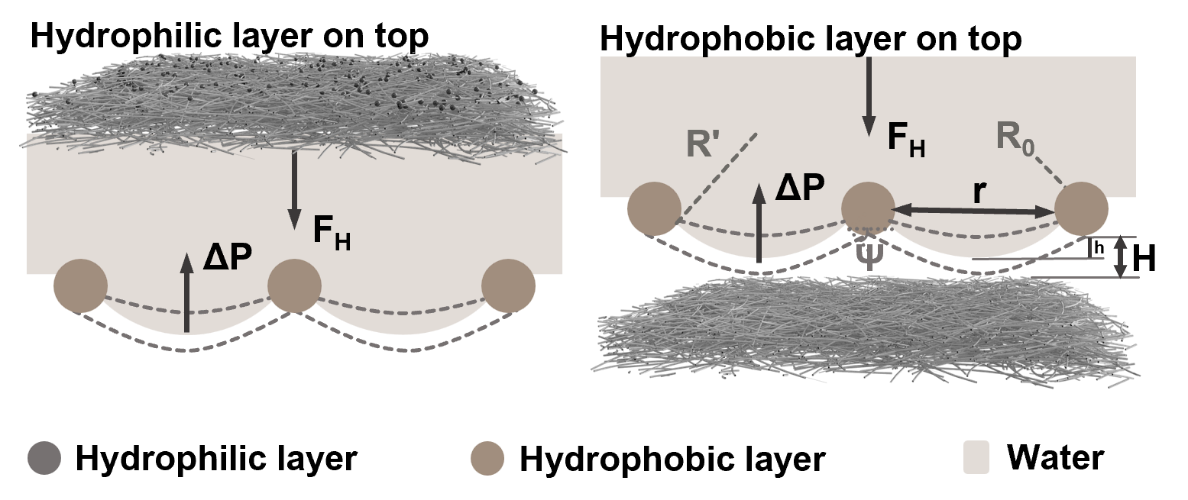


**Fig. S14** Proposed analysis model of the implementation of the directional moisture wicking in two situations


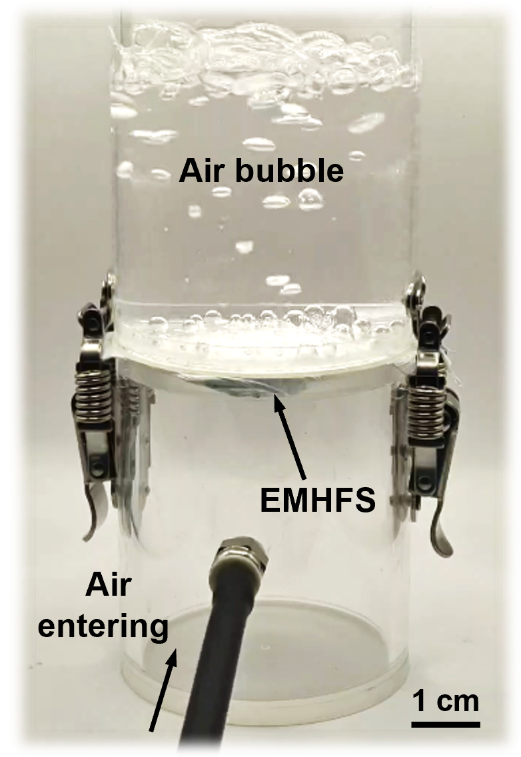


**Fig. S15** Digital image illustrating the breathability of EMHFS


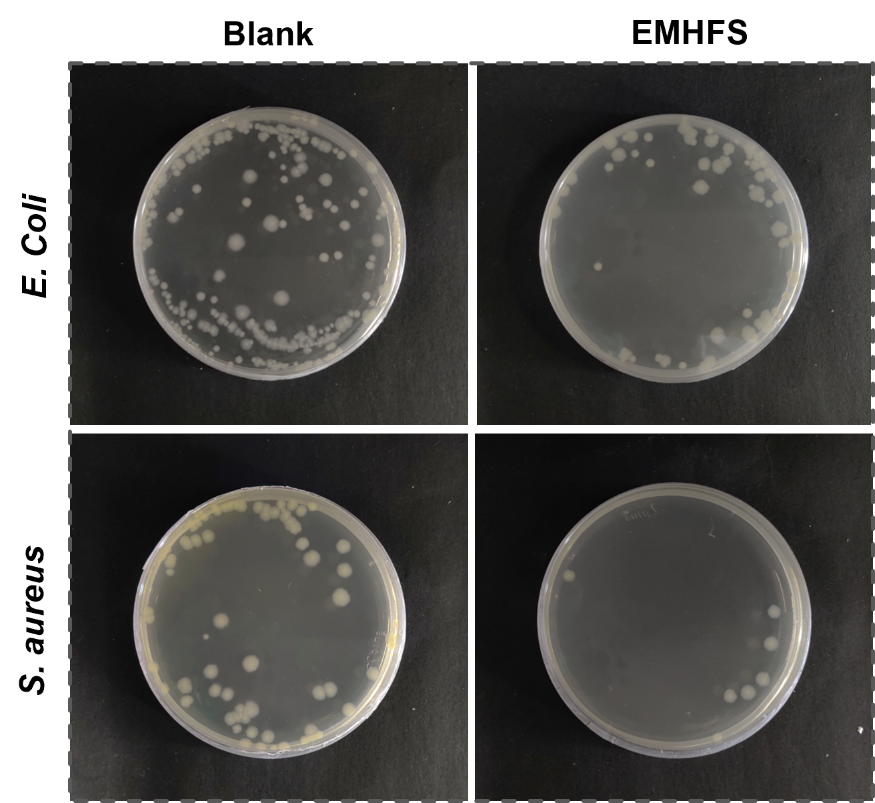


**Fig. S16** Digital photos of *E. coli* and *S. aureus* after co-culturing with blank and EMHFS


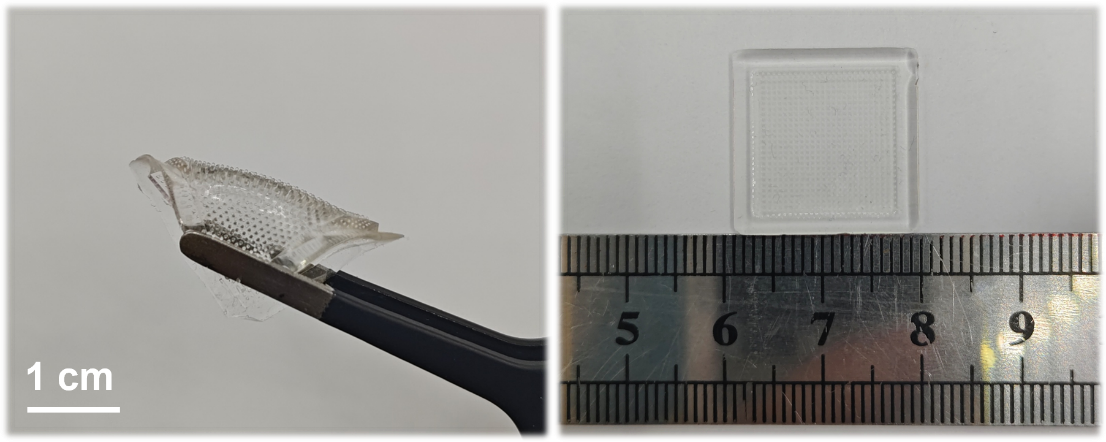


**Fig. S17** Physical photograph of the pure PDMS flexible substrate with papillary microstructures


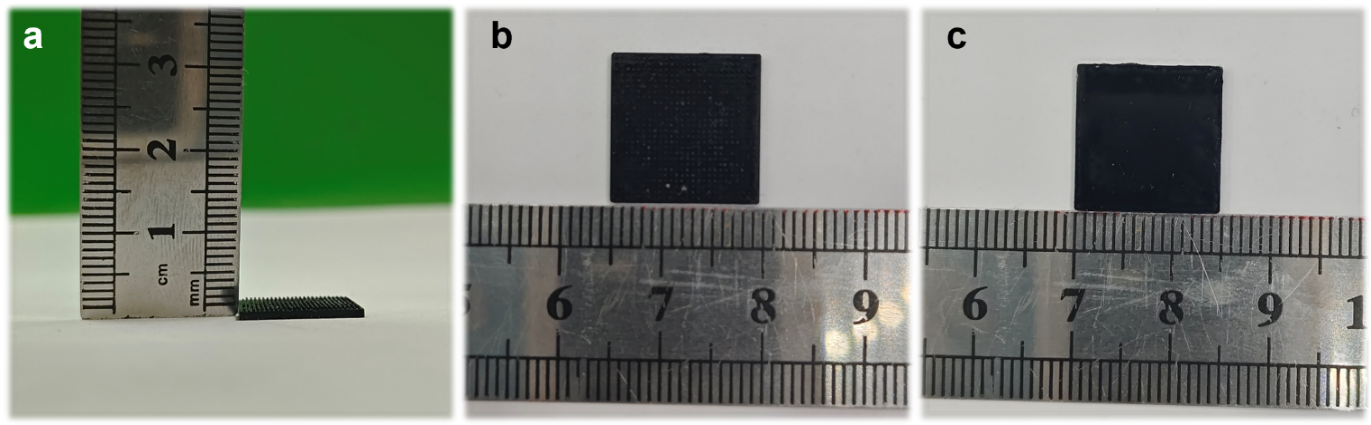


**Fig. S18** **a** Cross-sectional view, **b** front view, and **c** rear view of the piezoresistive layer with papillary microstructure after precision cutting

The piezoresistive layer was fabricated using polydimethylsiloxane (PDMS) as a flexible matrix and carbon nanotubes (CNTs) as the sensing material. The inherent flexibility and thermosetting properties of PDMS enable the formation of devices with diverse microstructures. As shown in Fig. S17, the pure PDMS substrate exhibits dimensions of 2 cm×2 cm after demolding, featuring an array of papillary microstructures. Following the incorporation of the conductive network, the demolded PDMS/CNTs composite is precisely cut into piezoresistive layers with uniform dimensions (1.5 cm×1.5 cm) and consistent thickness (approximately 1 mm) (Fig. S18).


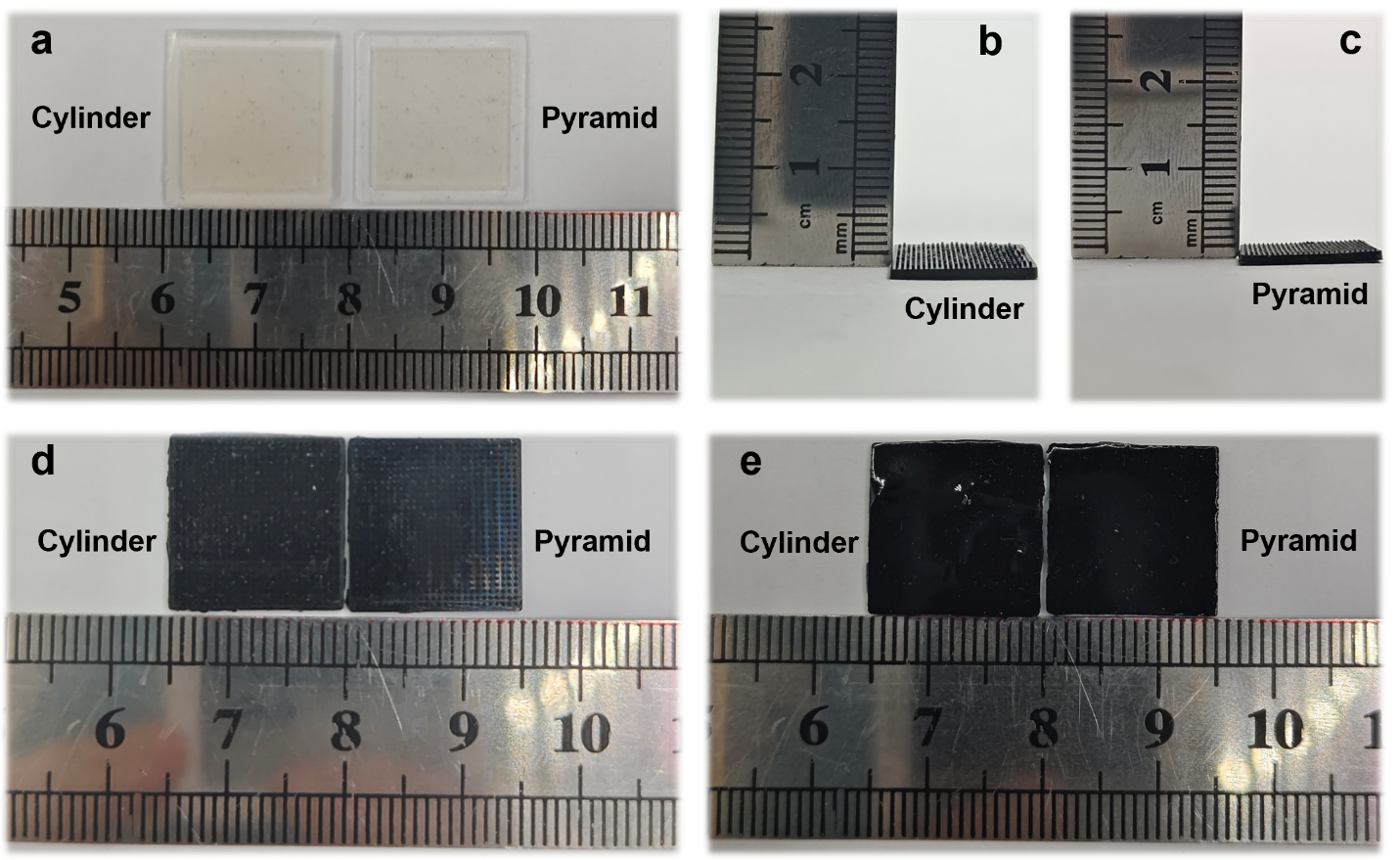


**Fig. S19** **a** Physical photograph of the pure PDMS flexible substrate with cylindrical and pyramidal microstructures. **b, c** Cross-sectional view, **d** front view, and **e** rear view of the piezoresistive layer with cylindrical and pyramidal microstructure after precision cutting


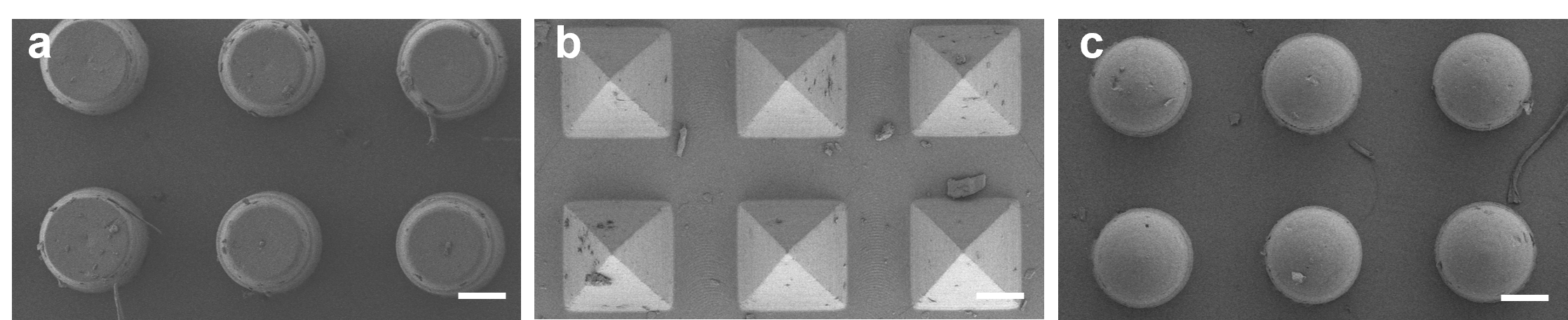


**Fig. S20** SEM images of piezoresistive layer with different microstructures: **a** cylinder, **b** pyramid, and **c** papilla. Scale bars, 100 μm


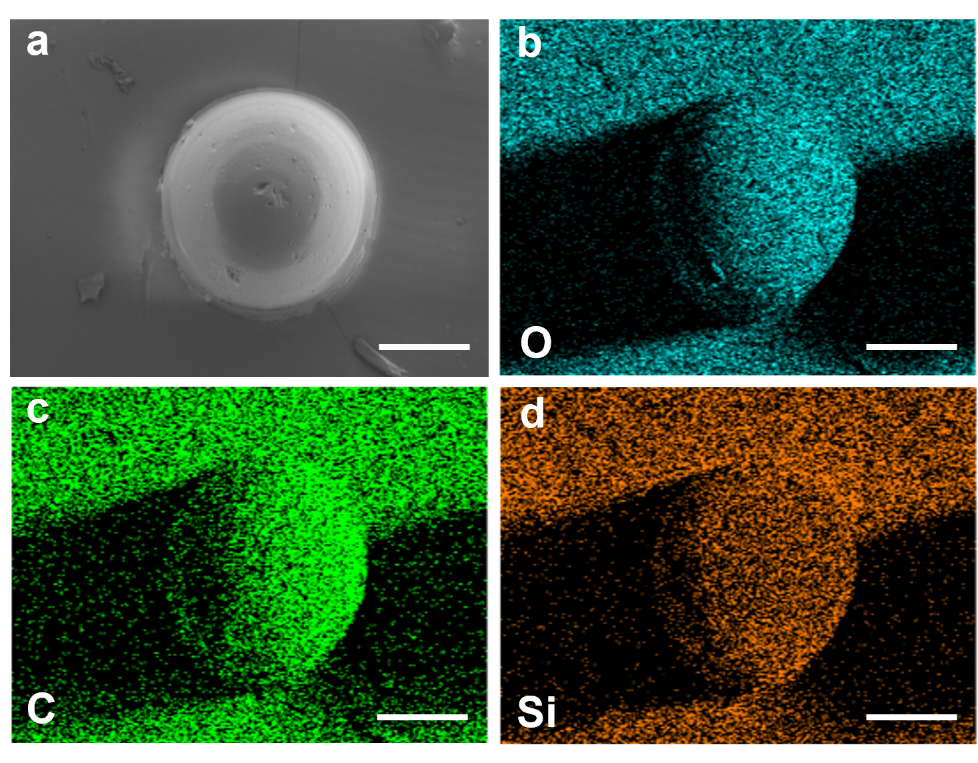


**Fig. S21** EDS mapping of **a** the papilla structure with element distribution images of **b** O, **c** C, and **d** Si. Scale bars, 100 μm


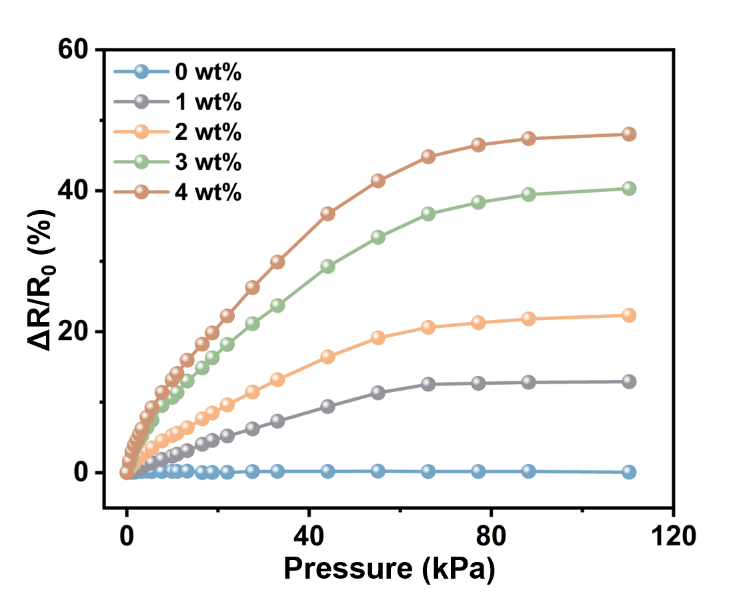


**Fig. S22** Pressure sensing performance of EMHFS with varying CNT contents


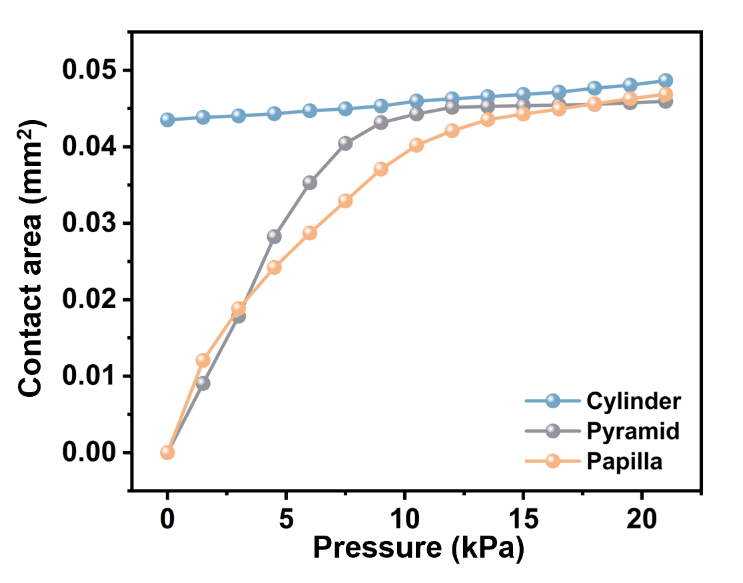


**Fig. S23** Variation curve of contact area between different microstructures and the electrode with pressure


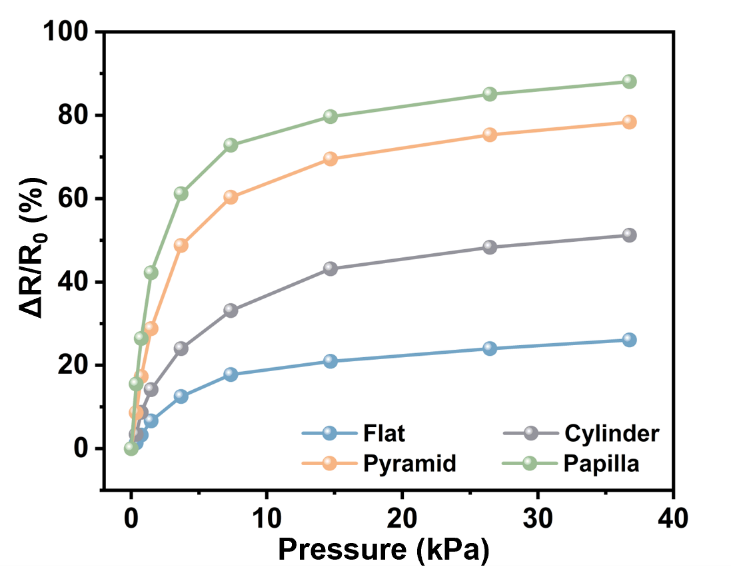


**Fig. S24** Pressure sensing performance of different structures


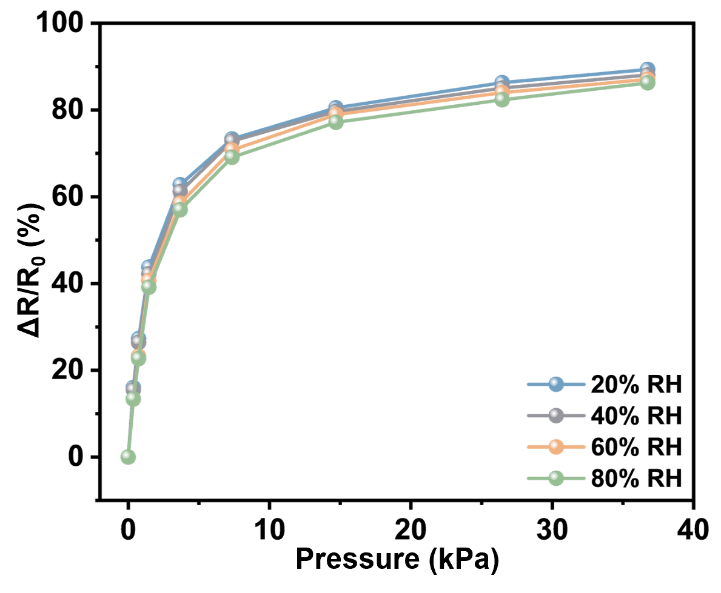


**Fig. S25** Pressure sensing performance of EMHFS at different relative humidity


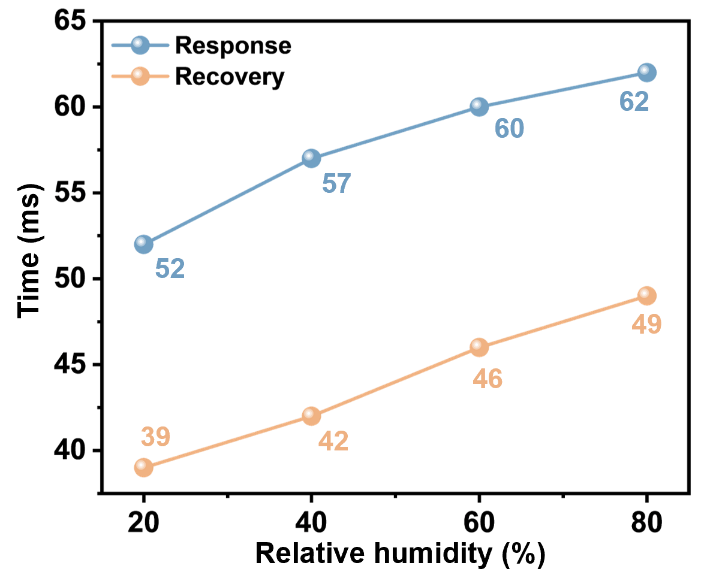


**Fig. S26** Response and recovery time of EMHFS under different relative humidity


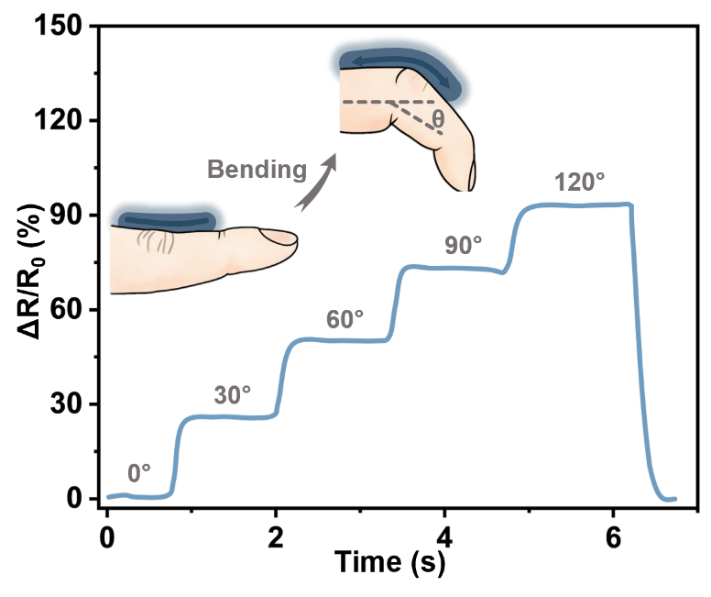


**Fig. S27** The step change of resistance when bending at different angles


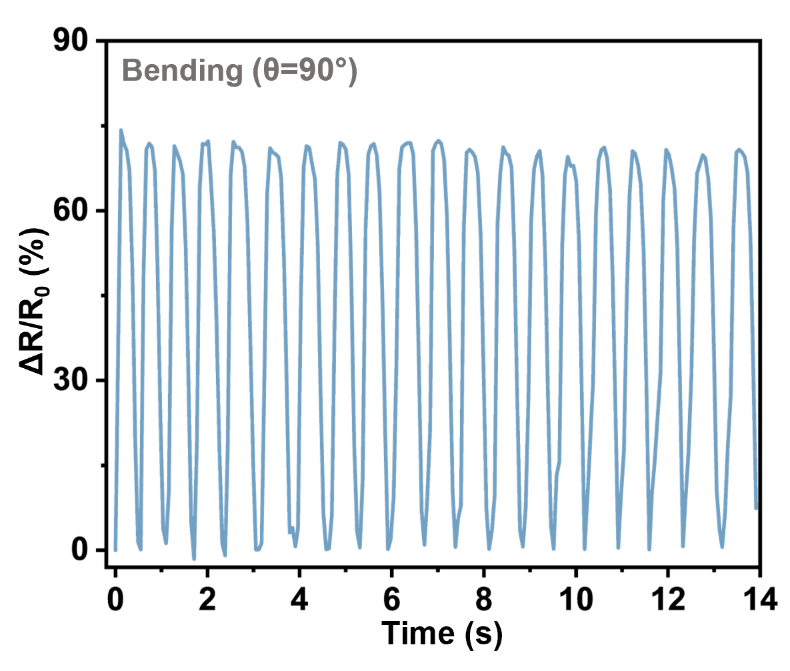


**Fig. S28** The resistance changes of EMHFS after 20 bends at 90°


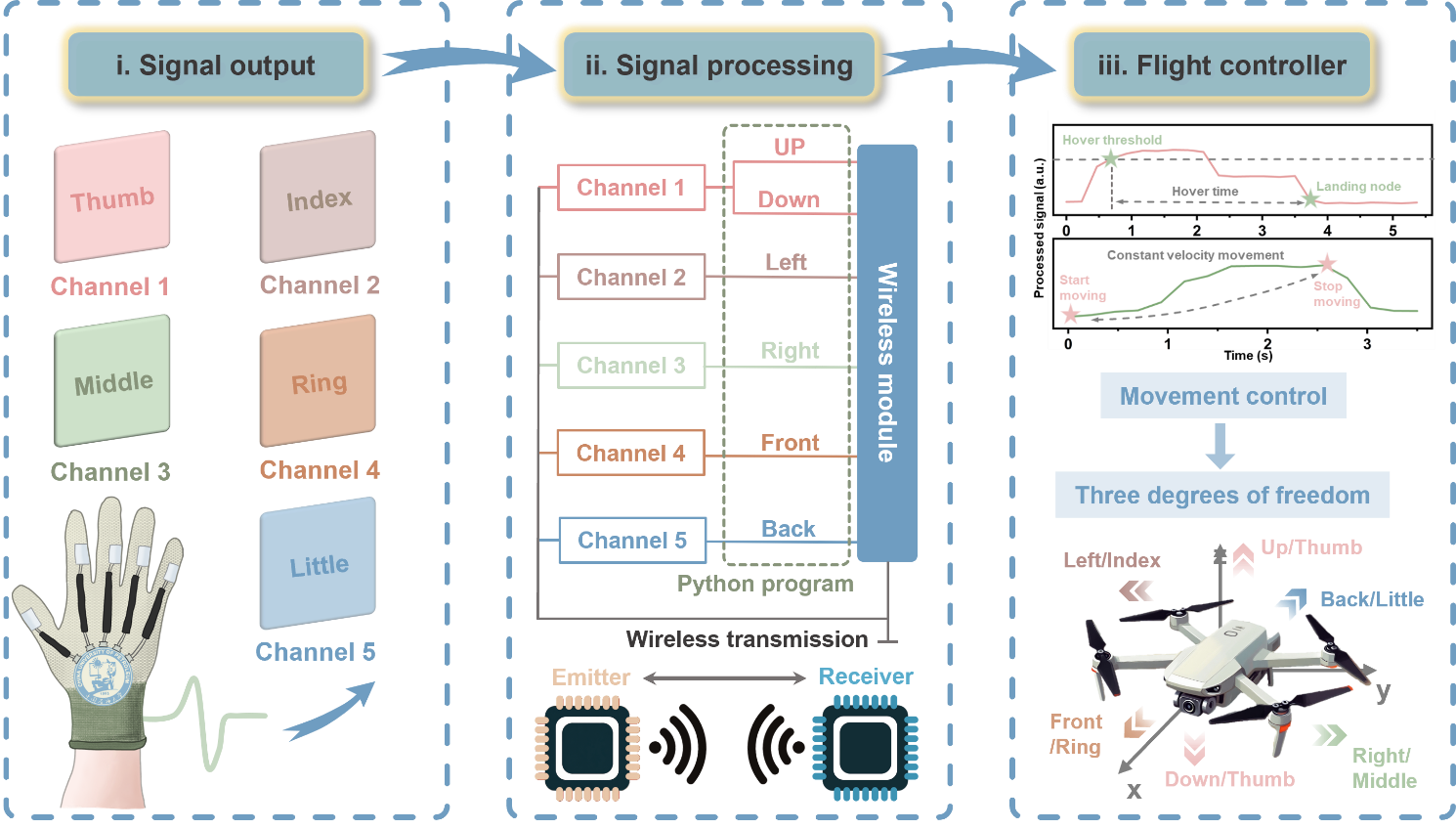


**Fig. S29** The schematic diagram depicts the principle of the proposed UAV control system.

Fig. S29 illustrates the operating principle of the proposed UAV control system, which consists of three main components: signal output, signal processing, and flight controller. 1) The thumb, index, middle, ring, and little fingers correspond to five independent signal channels (Channels 1-5), where the bending motion of each finger triggers an electrical output in its respective channel. 2) Each channel is assigned a specific flight action. Signals from all channels are parsed by a Python program into concrete action commands. These commands are transmitted wirelessly via a transmitter to the UAV receiver through a wireless module, enabling long-distance command delivery. 3) Upon receiving commands, the MCU on the UAV utilizes an integrated flight control module to regulate flight by adjusting motor speeds. This ultimately achieves stable control of the UAV's 3-DoF.


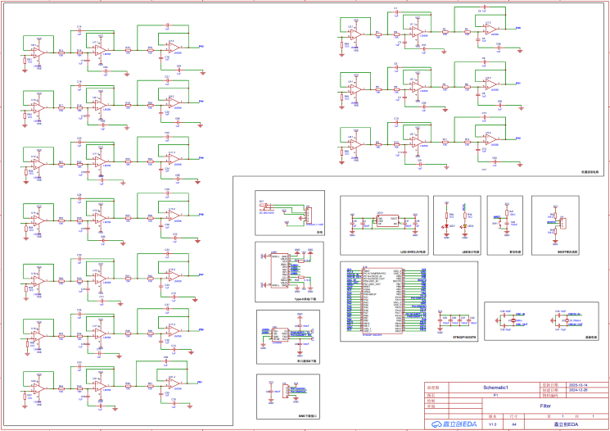


**Fig. S30** The circuit schematic of touchless screen control system for password and gesture unlocking


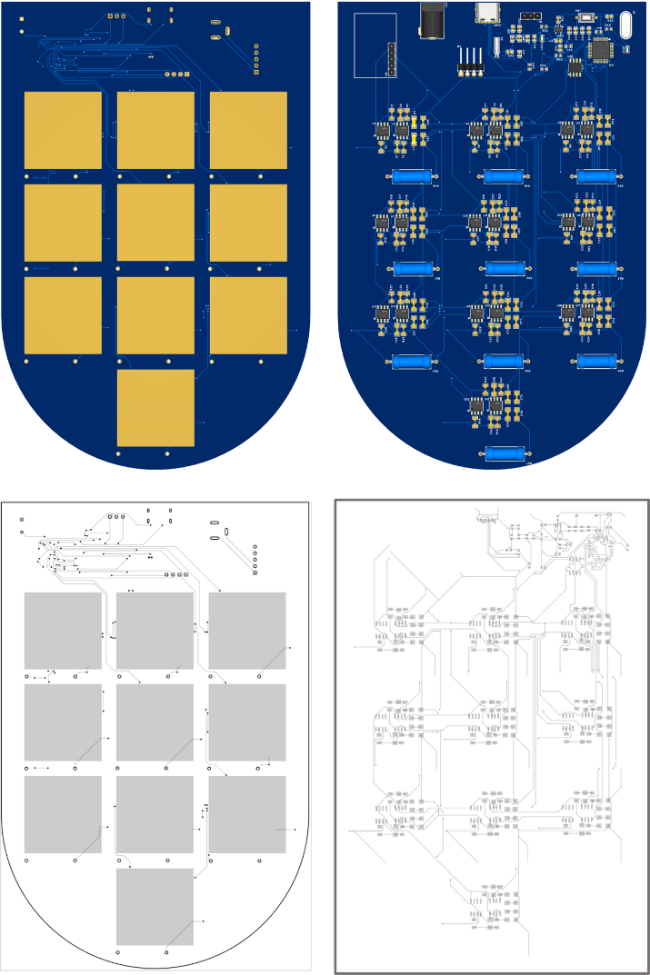


**Fig. S31** Top and bottom views of the PCB and corresponding wiring design


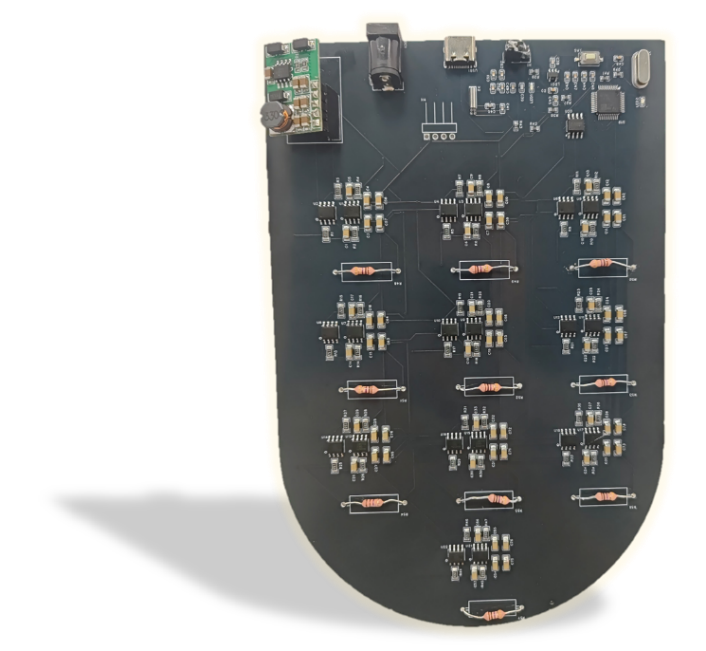


**Fig. S32** The physical image of PCB


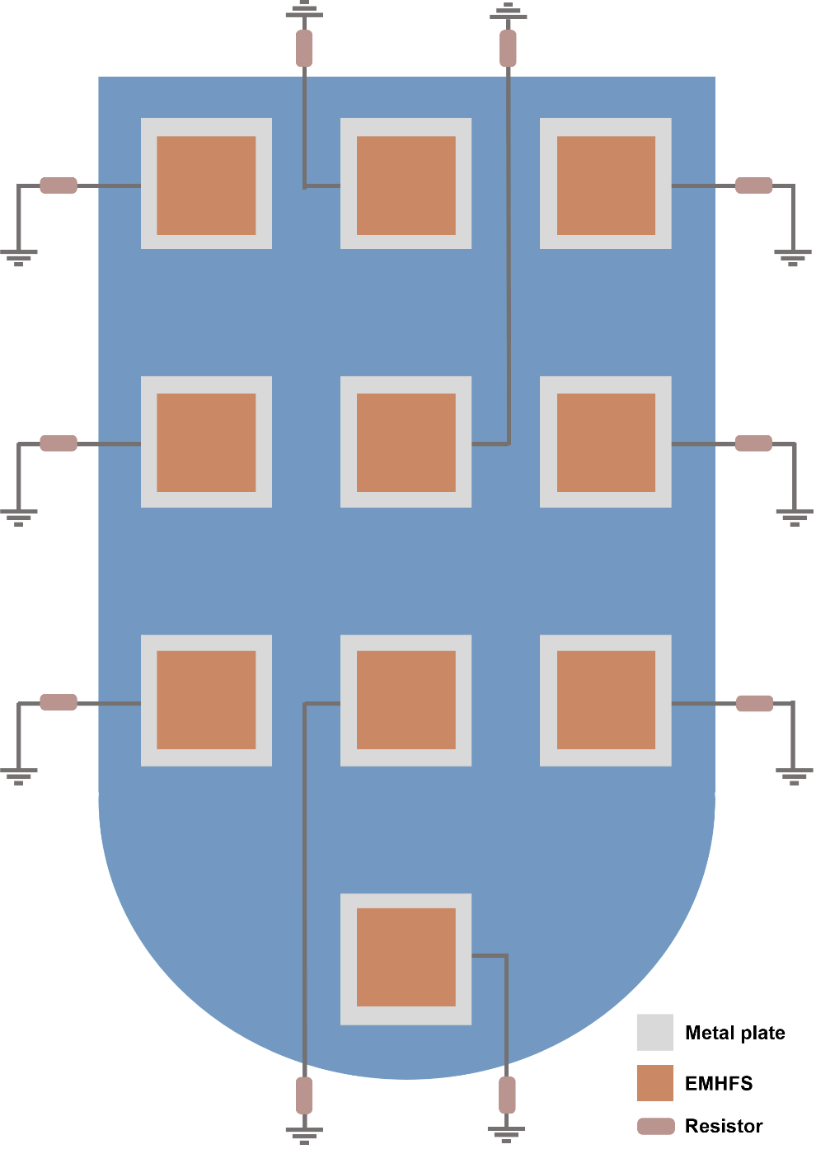


**Fig. S33** Schematic diagram of the equivalent circuit for the EMHFS sensor array used in touchless screen password and gesture unlock operations


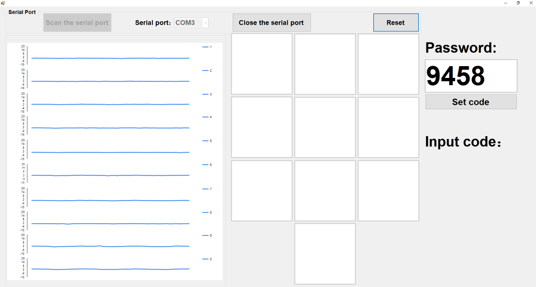


**Fig. S34** The initial interface of password unlocking for touchless screen control system


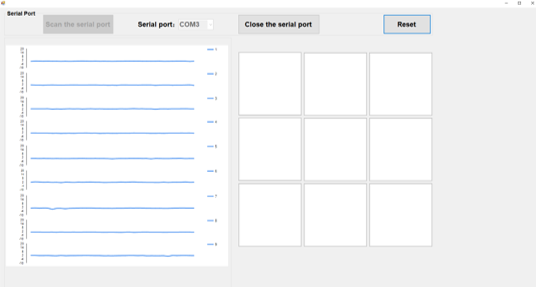


**Fig. S35** The initial interface of gesture unlocking for touchless screen control system

| **Table S1 The top-down fully bioinspired layered structure and functional description of EMHFS.** | | | |
| --- | --- | --- | --- |
| **Eggshell** | **EMHFS** | **Composition** | **Function** |
| Cuticle layer | Triboelectric layer | PVDF/MXene/BaTiO_3_ nanofibers | Noncontact sensing |
| Spongy layer | Piezoresistive layer | PDMS/CNTs composite | Pressure sensing |
| Papillary layer |  |  |  |
| Outer shell membrane | Hydrophilic layer | PAN nanofibers | Directional moisture wicking |
| Inner shell membrane | Hydrophobic layer | TPU nanofibers |  |

| **Table S2 Comparison of noncontact sensing between EHMFS and reported sensors.** | | | | |
| --- | --- | --- | --- | --- |
| **Material** | **Area (cm^2^)** | **Detection range (cm)** | **D/S (cm^-1^)** | **Refs.** |
| PTFE/Cu | 4×4 | 11 | 0.69 | [S2] |
| PDMS/Al | 7×3.6 | 150 | 5.95 | [S3] |
| PVDF@Ti_3_C_2_T_x_ composite film/Cu | 4×4 | 70 | 4.38 | [S4] |
| CCTO/Graphene | 2×2 | 1 | 0.25 | [S5] |
| Polyamide@Ag/PTFE/Cu | 6×6 | 18 | 0.5 | [S6] |
| Fish bladder film/Cu | 4×4 | 11 | 0.69 | [S7] |
| MXene/silicone | 8×4 | 200 | 6.25 | [S8] |
| FEP/PDMS/PET | 3×3 | 250 | 27.78 | [S9] |
| PI-b-C_60_/Al | 2×2 | 0.4 | 0.1 | [S10] |
| PVDF/MXene/BaTiO_3_ nanofibers/Ag | 1.5×1.5 | 200 | 88.89 | This work |

| **Table S3 Comparison of pressure sensing performances between EHMFS and reported sensors.** | | | | | | |
| --- | --- | --- | --- | --- | --- | --- |
| **Material** | **Sensitivity (kPa^-1^)** | **Detection range (kPa)** | **Response time (ms)** | **Recovery time (ms)** | **Cycles** | **Refs.** |
| Porous PDMS with groove | 0.18 | 50 | 52 | 60 | 1000 | [S11] |
| AgNWs/microneedle arrays | 0.203 | 60 | 125 | 250 | 2500 | [S12] |
| TPU/CNT foam | 0.309 | 80 | 40 | 80 | 600 | [S13] |
| CNT-wrapped PDMS microspheres | 0.111 | 50 | 200 | 150 | 12000 | [S14] |
| rGO/polyaniline sponge | 0.152 | 27 | 96 | - | 9000 | [S15] |
| MWNT–rGO-wrapped PU foams | 0.088 | 48.8 | 30 | - | 5000 | [S16] |
| (rGO@AgNPs)2@PPS | 0.0132 | 120 | 60 | 82 | 4000 | [S17] |
| TPU/MWCNT with microsphere | 0.285 | 77.93 | 50 | 100 | 1000 | [S18] |
| rGO@(PET-PU) | 0.255 | 108 | 313 | 318 | 1800 | [S19] |
| PDMS/CNTs with papilla | 0.287 | 105.8 | 57 | 42 | 13000 | This work |

**Supplementary Movies**

**Movie S1** Gesture-controlled robotic hand in real time.

**Movie S2** The EMHFS-based wearable UAV control system for 3-DoF stable control.

**Movie S3** EMHFS for password unlocking on touchless screen.

**Movie S4** EMHFS for "U" shape gesture unlocking on touchless screen.

**Movie S5** EMHFS for "L" shape gesture unlocking on touchless screen.

**Supplementary References**

1. P. Zhang, W. He, G. Wu, Y. Liu, X. Zhang et al., Hierarchically structured dual-mode sensor with directional moisture-wicking capability for weak physiological signal monitoring and long-range motion tracking. Chem. Eng. J. **520**, 165585 (2025). <https://doi.org/10.1016/j.cej.2025.165585>
2. H. Guo, X. Jia, L. Liu, X. Cao, N. Wang et al., Freestanding triboelectric nanogenerator enables noncontact motion-tracking and positioning. ACS Nano **12**(4), 3461–3467 (2018). <https://doi.org/10.1021/acsnano.8b00140>
3. D.V. Anaya, K. Zhan, L. Tao, C. Lee, M.R. Yuce et al., Contactless tracking of humans using non-contact triboelectric sensing technology: Enabling new assistive applications for the elderly and the visually impaired. Nano Energy **90**, 106486 (2021). <https://doi.org/10.1016/j.nanoen.2021.106486>
4. W. Zhang, Y. Lu, T. Liu, J. Zhao, Y. Liu et al., Spheres multiple physical network-based triboelectric materials for self-powered contactless sensing. Small **18**(25), 2200577 (2022). <https://doi.org/10.1002/smll.202200577>
5. S.A. Han, W. Seung, J.H. Kim, S.-W. Kim, Ultrathin noncontact-mode triboelectric nanogenerator triggered by giant dielectric material adaption. ACS Energy Lett., 1189–1197 (2021). <https://doi.org/10.1021/acsenergylett.0c02434>
6. L.-W. Lo, H. Shi, H. Wan, Z. Xu, X. Tan et al., Inkjet-printed soft resistive pressure sensor patch for wearable electronics applications. Adv. Mater. Technol. **5**(1), 1900717 (2020). <https://doi.org/10.1002/admt.201900717>
7. J. Ma, J. Zhu, P. Ma, Y. Jie, Z.L. Wang et al., Fish bladder film-based triboelectric nanogenerator for noncontact position monitoring. ACS Energy Lett. **5**(9), 3005–3011 (2020). <https://doi.org/10.1021/acsenergylett.0c01062>
8. C. Zhao, Z. Wang, Y. Wang, Z. Qian, Z. Tan et al., MXene-composite-enabled ultra-long-distance detection and highly sensitive self-powered noncontact triboelectric sensors and their applications in intelligent vehicle perception. Adv. Funct. Mater. **33**(52), 2306381 (2023). <https://doi.org/10.1002/adfm.202306381>
9. N. Dai, X. Guan, C. Lu, K. Zhang, S. Xu et al., A flexible self-powered noncontact sensor with an ultrawide sensing range for human-machine interactions in harsh environments. ACS Nano **17**(24), 24814–24825 (2023). <https://doi.org/10.1021/acsnano.3c05507>
10. J.W. Lee, S. Jung, J. Jo, G.H. Han, D.-M. Lee et al., Sustainable highly charged C60-functionalized polyimide in a non-contact mode triboelectric nanogenerator. Energy Environ. Sci. **14**(2), 1004–1015 (2021). <https://doi.org/10.1039/D0EE03057K>
11. H. Cui, Y. Liu, R. Tang, J. Ren, L. Yao et al., A sensitive and flexible capacitive pressure sensor based on a porous hollow hemisphere dielectric layer. Micromachines **14**(3), 662 (2023). <https://doi.org/10.3390/mi14030662>
12. C. Ni, S. Shang, J. Xu, M. Jin, H. Jiang et al., Flexible capacitive pressure sensor with optimized interdigital AgNWs electrodes for wide-range pressure monitoring. IEEE Sens. J. **24**(24), 40532–40540 (2024). <https://doi.org/10.1109/jsen.2024.3486089>
13. A. Huang, S. Gu, Z. Yang, X. Chen, M. He et al., Flexible, lightweight, and hydrophobic TPU/CNT nanocomposite foam with different surface microstructures for high-performance wearable piezoresistive sensors. J. Polym. Sci. **63**(3), 709–723 (2025). <https://doi.org/10.1002/pol.20240704>
14. M. Xu, Y. Gao, G. Yu, C. Lu, J. Tan et al., Flexible pressure sensor using carbon nanotube-wrapped polydimethylsiloxane microspheres for tactile sensing. Sens. Actuat. A Phys. **284**, 260–265 (2018). <https://doi.org/10.1016/j.sna.2018.10.040>
15. G. Ge, Y. Cai, Q. Dong, Y. Zhang, J. Shao et al., A flexible pressure sensor based on rGO/polyaniline wrapped sponge with tunable sensitivity for human motion detection. Nanoscale **10**(21), 10033–10040 (2018). <https://doi.org/10.1039/C8NR02813C>
16. A. Tewari, S. Gandla, S. Bohm, C.R. McNeill, D. Gupta, Highly exfoliated MWNT–rGO ink-wrapped polyurethane foam for piezoresistive pressure sensor applications. ACS Appl. Mater. Interfaces **10**(6), 5185–5195 (2018). <https://doi.org/10.1021/acsami.7b15252>
17. [17] B. Chen, H. Li, S. Zhang, X. Lai, X. Zeng et al., High-performance and superhydrophobic piezoresistive pressure sensor based on mountain ridge-like microstructure by silver nanoparticles and reduced graphene oxide. Compos. Part A Appl. Sci. Manuf. **162**, 107171 (2022). <https://doi.org/10.1016/j.compositesa.2022.107171>
18. A. Huang, Z. Yang, Y. Zhu, B. Tan, Y. Song et al., A unique, flexible, and porous pressure sensor with enhanced sensitivity and durability by synergy of surface microstructure and supercritical fluid foaming. Appl. Surf. Sci. **618**, 156661 (2023). <https://doi.org/10.1016/j.apsusc.2023.156661>
19. H. Cheng, N. Zhang, Y. Yin, C. Wang, A high–performance flexible piezoresistive pressure sensor features an integrated design of conductive fabric electrode and polyurethane sponge. Macromol. Mater. Eng. **306**(9), 2100263 (2021). <https://doi.org/10.1002/mame.202100263>
